# The road to negation: A comparative study of five typologically and culturally diverse languages

**DOI:** 10.1177/01427237251336806

**Published:** 2025-06-19

**Authors:** Sakine Çabuk-Ballı, Aylin C Küntay, Paul Widmer, Sabine Stoll

**Affiliations:** Institute for the Interdisciplinary Study of Language Evolution, University of Zurich, Switzerland; Department of Psychology, Koç University, Turkey; Institute for the Interdisciplinary Study of Language Evolution, University of Zurich, Switzerland; Institute for the Interdisciplinary Study of Language Evolution, University of Zurich, Switzerland

**Keywords:** Negative functions, acquisition, child-directed language, cross-linguistic, input, sentence type

## Abstract

The acquisition of negation is a key milestone in early language development that enables children to express rejection, non-existence, and deny propositions. In this study, we ask whether the development of the functions of negation follows a universal trajectory or varies based on language-specific features and environmental input. We investigate the acquisition of negative functions in 10 children (age = 2;00-3;00) across five typologically maximally diverse languages: Chintang (Sino-Tibetan), English (Indo-European), Indonesian (Austronesian), Sesotho (Bantu), and Turkish (Turkic). Our findings reveal language-specific developmental trajectories within our sample. Notable differences include variations in the frequency of *denial* in child speech and *prohibition* in the ambient language. A strong correlation emerged between the presence of these negative functions in child-directed speech and their use by children. Across the five languages, children pre-dominantly use declarative negative sentences and begin gradually incorporating imperative forms into their repertoire of negative utterances over time. The emergence of negative questions occurs towards the end of age 2, but remains a minor component of children’s negative expressions. The overall pattern observed in our maximum diversity sample highlights the significant role of child-directed input and cross-cultural variation in shaping the developmental trajectory of negative functions.

## Introduction

Negation constitutes a fundamental and ubiquitous feature of human languages (Dahl, 1979; Horn, 1989; Jespersen, 1917). It serves as a crucial mechanism that enables humans to reason, interpret, and communicate a broad range of ideas across diverse contexts. It allows speakers to express disagreement, refusal, sarcasm, skepticism, and a variety of other communicative functions. Although the conceptual foundation of negation is often assumed to be cognitively universal, its linguistic encoding varies considerably across languages, which potentially has an impact on the learning of communicative negative functions. The present study focuses on linguistic expressions of negation, rather than its conceptual representation. It aims to clarify how language-specific forms of negation influence the development of negative functions in child language acquisition in a set of typologically diverse languages.

Negation has long been a focus in research on child language development. At a very early age, children start out by using non-verbal forms of negation, such as rejecting objects through hand gestures or head shakes. These non-verbal expressions of negation typically emerge in the latter half of the first year of life, well before children develop the ability to express negation verbally (Morgenstern et al., 2018). As children’s speech develops, gestures are gradually paired with verbal expressions of negation. This integration of gestural and verbal modalities reflects their growing ability to use language more flexibly and efficiently to communicate meanings beyond the immediate physical rejection of objects (Morgenstern et al., 2016). However, gestures remain essential communicative tools, particularly in contexts where they enhance or clarify meaning (Beaupoil-Hourdel et al., 2016; Morgenstern et al., 2018).

It is assumed that in the early stages of language development, the expression of negation by young children encompasses only a small fraction of the range of functions observed in adult speech. This limitation is largely attributed to the extraordinary challenge of integrating negation into the linguistic system, which develops gradually over time (Çabuk-Ballı et al., 2025; Feiman et al., 2017; Hochmann, 2020), and the limited availability of cognitive resources during early development (Austin, et al., 2014; Bloom, 1970; Nordmeyer & Frank, 2014). In early acquisition, the word *no*, or its equivalent, is among the earliest words produced by children (e.g. Mother: *Do you want some milk?* Child: *No*) (Braginsky et al., 2016; Schneider et al., 2015). Although this may seem simple from a production standpoint, using *no* appropriately requires understanding the entire proposition made by the interlocutor. In fact, unlike affirmative responses and statements, negation introduces an additional layer of abstraction, which requires children to process not only the affirmative aspects of a situation but also how negation alters the truth-functional value of a proposition (Szabó & Kovács, 2025). Infants may possess an early conceptual understanding of negation but struggle with mapping this concept onto specific linguistic forms or processing more complex types of negation (Gomes et al., 2023; Nordmeyer & Frank, 2014; Szabó & Kovács, 2025).

Earlier research on the development of negative functions primarily focused on the timeline and mechanisms behind the emergence of various forms and meanings of negation in children’s language (e.g. Bloom, 1973; R. Brown, 1973; Pea, 1980; Wode, 1977). Different taxonomies were proposed to categorize functions of negation in infants and toddlers, typically encompassing rejection, prohibition, failure, denial, and non-existence, or a subset of these (Bloom, 1970; Choi, 1988). In this line of research, diverse developmental patterns were found: rejection appears first, followed by non-existence and finally denial in English and Tamil (Pea, 1980; Vaidyanathan, 1991), whereas in Hong Kong Cantonese and several other languages, non-existence precedes rejection (Choi, 1988; Tam & Stokes, 2001). Despite the differences in the exact order reported across studies, rejection and non-existence consistently emerge earlier than denial or truth-functional negation (Bloom, 1970; Choi, 1988; Pea, 1982). Some studies expanded the scope to include gestural expressions and single-word utterances, and identified a developmental sequence in which rejection appears first, followed by self-prohibition, disappearance, unfulfilled expectation, and finally, truth-functional negation (Pea, 1980).

These findings from previous research suggest that infants’ early understanding of negation is initially confined to a limited range of meanings, primarily centered on situation-bound concepts such as rejection (e.g. refusing an object or action) or non-existence (e.g. recognizing the absence of something). These early expressions of negation often emerge when an infant’s expectations are violated (e.g. when an object disappears) or when their desires are unmet (e.g. pushing something away) (Austin et al., 2014). In this view, children gradually expand their understanding of negation as they mature and eventually develop a more comprehensive and abstract grasp of negation that encompasses a wider array of functions, such as denial, inability, and inferential negation. This developmental trajectory suggests that children initially adopt a simple, context-bound interpretation of negation, closely linked to their immediate experiences and the presence or absence of desired objects or actions. Only later do more abstract functions of negation, such as denial and epistemic negation (i.e. expressing doubt or uncertainty, as in *I do not know* or *It may not be true*), emerge in their cognitive and linguistic repertoire (Austin et al., 2014; Choi, 1988; Hummer et al., 1993). Contrary to earlier assumptions, more recent studies suggest that the developmental trajectory of negation might not be as consistent as previously thought (Liu & Jasbi, 2025; Nordmeyer & Frank, 2018). English-speaking children were found to use *denial* at an earlier age and more frequently than previously reported in the literature (Nordmeyer & Frank, 2018). Furthermore, large-scale studies on the development of negation in English find no strong evidence for population-level stages; however, children’s ability to use different constructions to express various functions of negation begins to emerge around 18 to 22 months (Liu & Jasbi, 2025).

Two complementary perspectives were proposed to explain the apparent discrepancy between infants’ early production of negation and their later success in comprehension tasks. One view suggests that infants possess a conceptual understanding of negation from an early age, but their performance in comprehension tasks is hindered by general language processing difficulties (Feiman et al., 2017). These challenges include mapping abstract negation concepts to linguistic forms and processing complex sentence structures. Rather than emerging in tandem with other cognitive milestones, negation may be an abstract concept that infants grasp early on but struggle to map onto specific linguistic forms or processes within more complex syntactic structures (Gomes et al., 2023; Szabó & Kovács, 2025). The term abstract concept is often ambiguous, but one possible interpretation is that negation does not correspond directly to a concrete object or action; instead, it involves a mental representation of absence, opposition, and denial. Studies indicate that infants struggle even with affirmative statements, and their difficulties with negation may stem from broader sentence-processing constraints rather than a lack of conceptual competence (Austin et al., 2014; Reuter et al., 2018). For instance, to understand ‘*It is not in the basket*’, infants must grasp negation and the process of the complex proposition it modifies along with its elements. Furthermore, evidence from non-verbal reasoning tasks supports the idea that infants can engage in exclusion-based reasoning, which may serve as a precursor to linguistic negation (Cesana-Arlotti et al., 2018, 2020; Dudley et al., 2023). The difficulty with negation may arise because negation is not merely a conceptual operation but a linguistic one that requires integration into a broader grammatical system, which requires mastering language-specific syntactic structures and morphological markers. Beyond the inherent challenge of understanding truth-functional negation, the acquisition process is further influenced by the diverse ways in which different languages encode negation at various levels of grammatical complexity (Çabuk-Ballı et al., 2025; Dryer, 1988; Horn, 2010). These structural and functional variations introduce additional difficulties, making negation particularly challenging for early language learners (Dimroth, 2010; Horn, 2010; Szabó & Kovács, 2025).

An alternative account posits that infants’ early production of negation does not necessarily indicate a fully developed understanding of the concept. Instead, they may initially use negation in a limited, context-dependent manner. According to this perspective, early utterances like *all gone* may not represent true propositional negation but rather simple absence-related expressions (Cameron-Faulkner et al., 2007; Feiman et al., 2017). The emergence of logical operators and compositional thought is assumed to develop later, in close connection with linguistic experience (Mody & Carey, 2016). This view aligns with theories suggesting that children initially learn multi-word utterances as fixed phrases and only gradually begin to use them in abstract, flexible ways (Lieven et al., 1997; Tomasello, 2000). In English, for example, children must learn to use negation markers such as *not* or contractions such as *don’t*, master their syntactic positioning, and understand their interactions with other elements in a sentence (Cameron-Faulkner et al., 2007). Additionally, children must associate negation markers with different functions, such as prohibition, denial, and epistemic negation, while also understanding how these functions interact with syntactic structures and discourse contexts (Cameron-Faulkner et al., 2007; Grigoroglou & Ganea, 2022). This learning process entails not only acquiring the correct placement and form of negation markers but also understanding their pragmatic roles, which may vary across languages. This perspective challenges the notion that negation emerges in tandem with other cognitive milestones, such as theory of mind and object permanence (Bloom, 1970; Choi, 1988; Feiman et al., 2017; Pea, 1982). Instead, it suggests that while infants may possess a conceptual understanding of negation from an early age, their primary difficulty lies in mastering the intricate linguistic and grammatical structures necessary for its full expression (Austin et al., 2014; Reuter et al., 2018).

While existing research offers important insights into the development of negative functions in child speech, several areas remain underexplored. First, the role of child-directed speech (CDS) is usually given only limited attention, despite its crucial influence on how children acquire and use negation. The structure, frequency, and form of negation in CDS presumably influence the child’s ability to recognize and reproduce different negative functions. Repetition and emphasis, which are typical of CDS, can provide essential cues that help children understand how negation functions in various contexts (Nordmeyer & Frank, 2018). Second, research on the acquisition of negation has mainly focused on a limited set of Western Indo-European languages, such as English (e.g. Cameron-Faulkner et al., 2007; Liu & Jasbi, 2025; Nordmeyer & Frank, 2014), French (e.g. Meisel, 1997), German (e.g. Clahsen et al., 1993; Mills, 2017), Italian (e.g. Tagliani et al., 2022), Spanish (Bel, 1996). In contrast, studies examining negation in other linguistic and cultural settings remain comparatively scarce (e.g. Binturki, 2015; Choi, 1988; Hummer et al., 1993; White et al., 2023). Whether existing findings generalize beyond this narrow typological range is still an open question. Third, little is known about the specific sentence structures in which different negative functions first appear. This gap is significant, as sentence types define not only the structural realization of negation but also its pragmatic functions. The choice of a sentence type reflects the speaker’s communicative intent and the social context in which the utterance is produced (Goodhue et al., 2023). In addition, syntactic complexity impacts the linguistic expression of negation by determining how negation integrates into different sentence structures. For instance, imperatives require minimal syntactic adjustment, while more complex structures, such as interrogatives or subordinate clauses, impose greater cognitive and linguistic demands. As these structures are acquired, children must learn to integrate negation into increasingly complex syntactic frames. This progression necessitates the integration of syntactic, semantic, and pragmatic knowledge. Examining how negation operates within these structures can reveal how specific negative functions emerge as language development progresses.

To fill these gaps and make more general claims, we adopt a systematic cross-linguistic approach, selecting languages that differ maximally in their grammatical features (Stoll & Bickel, 2013). We investigate how children from diverse linguistic and cultural backgrounds learn to express different negative functions across different sentence types by analyzing both child speech/productions and the input children receive as well as the effect of CDS compared to child-surrounding speech on their development. Our analysis spans five cultural settings, including both western and subsistence communities: Chintang (Sino-Tibetan, Nepal), English (Indo-European, England), Indonesian (Austronesian, Indonesia), Sesotho (Bantu, Lesotho), and Turkish (Turkic, Turkey). We compare the development of negative functions in child speech relative to adults/the ambient language within the age range of 2 to 3 in naturalistic longitudinal corpora. We introduce sentence type as an additional dimension of variation and specifically ask: (a) How do the different negative functions develop over time across five linguistically and culturally diverse settings?, (b) To what extent does CDS in negative functions, compared to child-surrounding speech, contribute to the development of language-specific developmental trajectories?, (c) How do the different functions relate to the sentence type (declarative, imperative, interrogative, exclamative)?

Based on previous findings, we test whether negative functions are typically acquired in a generally consistent sequence (Choi, 1988; Pea, 1980), starting with more basic communicative functions (e.g. rejection, non-existence) and progressing towards more abstract negative functions (e.g. denial, epistemic negation). We expect significant variation in developmental trajectories across languages, driven by contextual factors such as the nature of parent-child interactions, cultural practices, and the specific linguistic structures to which children are exposed. With respect to CDS, we expect that the quantity of negative utterances directed at children may play a significant role in shaping their development of negative functions. Specifically, higher exposure to child-directed negation (e.g. prohibition or denial) may facilitate earlier acquisition of these forms, while environments that prioritize indirect negation (i.e. negative utterances heard in surrounding speech) could delay the development of specific negative functions (e.g. denial or epistemic negation). Furthermore, the frequency and contexts in which caregivers use negative utterances may affect the development of more complex and diverse negative forms in children. Regarding sentence types, we predict that children will first employ negation in simple declarative statements and, over time, incorporate imperatives and questions into their linguistic repertoire. This expectation is grounded in previous research (e.g. Dąbrowska & Lieven, 2005; Goodhue et al., 2023; Zaitsu et al., 2021).

## Methods

### Data

Our study is based on longitudinal audio-visual child language acquisition corpora from the ACQDIV Database (Moran et al., 2016) of typologically maximally diverse languages. The underlying rationale of this database conceptualized by Stoll and Bickel (2013) is to capture the linguistic diversity in the languages of the world using different clusters of languages that differ maximally in their grammatical structures. To detect these clusters, Stoll and Bickel (2013) employed a fuzzy clustering algorithm (Kaufman & Rousseeuw, 2009) in two large typological databases (AUTOTYP (Bickel et al. 2017) and WALS (Dryer&Haspelmath, 2013)) to maximize typological differences between languages with respect to a number of central typological parameters such as word order, inflectional classes, presence and nature of agreement, case marking, and degree of synthesis.

For the present study, we selected five languages from the ACQDIV Database: Chintang (Stoll et al., 2012), English (Manchester Corpus; Theakston et al., 2001), Indonesian (Indonesian Jakarta Corpus; Gil & Tadmor, 2007), Sesotho (Sesotho Demuth Corpus; Demuth, 2015), and Turkish (Koç University Longitudinal Language Development Database; Küntay et al. 2015). The languages are from different clusters of maximally diverse languages (Stoll & Bickel, 2013). To explore the development of negative functions in child speech relative to the input in the ambient language, we examined the recordings from 2 children for each language (a total of 10 children) between the ages of 2 and 3. This developmental stage marks the point at which negative expressions start to manifest themselves in various forms and contexts. The age range is segmented into monthly intervals (age bins) to explore the development over time. The recordings for English, Indonesian, and Sesotho are audio-only, while the recordings for Chintang and Turkish include both audio and video. The recordings were collected in different contexts and reflect diverse ecological and cultural settings. In our language sample, English, Turkish, and Indonesian (the recordings were conducted in Jakarta) reflect westernized and urban socio-cultural environments, while Chintang and Sesotho represent rural cultures. Table 1 provides some genealogical, demographic, and cultural information about the languages investigated in this study. In the westernized contexts, children receive direct input mainly from their caregivers, typically mothers and close family members (e.g. English), while in the recordings conducted in rural areas, children are exposed to input from a larger variety of sources, including other children and a variety of surrounding speakers (e.g. Chintang). The number of surrounding speakers in the recordings thus differs widely across languages/cultures. In the case of Chintang, children are exposed to a range of 4 to 12 surrounding speakers during their daily interactions (Stoll et al., 2012), while in other languages this number is comparatively lower (Table 2). The cultural context of language exposure can significantly influence the trajectory of language acquisition, as different social interactions and input dynamics shape the learning process (Bergelson et al., 2019; Casillas et al., 2020; Cristia et al., 2019). To understand how this influences the development of negative functions, we coded negative functions children hear in the ambient language as child-directed versus child-surrounding.

**Table 1.** Language sample with genealogical and demographic characteristics.

Language	Language family	*N* of speakers	Socio-economic status
Chintang	Sino-Tibetan	5–6 K	Subsistence, village
English	Indo-European	360 M	Industrialized, urban, middle-class
Indonesian	Austronesian	20 M	Urban, lower, and middle-class
Sesotho	Bantu	5.6 M	Subsistence, village
Turkish	Turkic	80 M	Urban, lower-middle class

**Table 2.** Number of words produced by children and surrounding speakers with typical MLU in words in child speech and number of surrounding speakers in the recording contexts.

Corpus	Words overall	*N* (words)		MLU (child)	*N* (Surrounding speakers)
		Child	Surrounding speakers		
Chintang	1,621,155	237,913	1,383,242	2.0	4–12
English	2,293,100	617,359	1,675,741	2.2	2–3
Indonesian	2,790,279	894,210	1,896,069	2.6	3–5
Sesotho	330,026	124,763	205,263	2.3	3–5
Turkish	1,233,221	215,394	1,017,827	2.4	3–6

*Note*. MLU = mean length of utterance.

Recording schemes reflect diverse contexts and distinct practices of linguistic communities. Consequently, they encompass variations in the quality and quantity of speech directed towards children. See Table 2 for some summary statistics of the corpora used.

The mean length of utterance (MLU) in words at the onset of age two was similar across children in our sample (MLU = 2.0; 2.6). There is a wide range of demographic differences in our sample, including socio-economic status and cultural background. However, in a longitudinal study, it is not feasible to strictly control for all these variables. We have balanced gender and maintained a focus on the relevant age range of interest. The socio-economic status of children learning these five languages varies significantly. In Chintang (Sino-Tibetan) and Sesotho (Bantu) communities, children are typically raised in subsistence-based, rural village settings. In contrast, our English (Indo-European) sample comes from urban, and predominantly middle-class environments. Our Indonesian (Austronesian) speakers come from diverse strata living in Jakarta. Similarly, our Turkish (Turkic) participants primarily live in a large city (Istanbul), with children from lower-middle and middle-class families.

### Classification of functions and data coding

#### Classification of negative functions

We distinguished eight types of negative functions in child and CDS, based on established categories proposed in the literature (e.g. Cameron-Faulkner et al., 2007; Choi, 1988). We further coded for sentence type to comprehensively capture the development of different negative functions in children’s linguistic systems between the ages of 2 and 3.

Negative functions summarized in Table 3 serve distinct purposes in communication. *Rejection* enables speakers to convey disagreement or disapproval by negating a proposition or an action (e.g. *I don’t want it*). It is widely regarded as one of the first negative meanings to emerge in child speech (Beaupoil-Hourdel et al., 2016; Bloom, 1973; Pea, 1980). This ability represents an important milestone in children’s linguistic and cognitive development as it allows children to articulate their desires and needs through rejection. Non-existence refers to the expression of an entity’s absence within a specific context (e.g. *There is no apple*). Through the concept of non-existence, children begin to grasp the distinction between the presence and absence of an entity, thereby enhancing their perception and comprehension of the world around them (Pea, 1980). *Denial* involves refuting the truth-functional value of a previous proposition (e.g. *It is not a cat; it’s a doggie*). Children begin to develop the ability to question or contradict information they perceive as false or inconsistent with their own experiences or beliefs with denial (Déprez & Espinal, 2020). *Prohibition* communicates rules or restrictions by forbidding certain actions or activities (e.g. *Don’t touch that!*). Prohibitive utterances are an integral part in the surrounding speech to guide children’s behaviors. Failure acknowledges the lack of success or achievement, and it becomes an essential component of children’s linguistic repertoire as they learn to articulate and discuss their experiences of not meeting desired outcomes or goals (e.g. *This doesn’t go in there*) (Bloom, 1970). Inability plays a vital role in the development of children’s linguistic repertoire as it enables them to communicate their limitations or the absence of physical capability in engaging in certain activities or tasks (e.g. *I can’t open it*). Epistemic negation denotes the absence of knowledge or understanding about a specific subject, and indicates that there is no evidence that the event is true (e.g. *I don’t know*). It becomes an integral part of children’s linguistic development as they learn to express uncertainty or lack of information.

**Table 3.** Classification of negative functions in child and child surrounding speech.

Category	Description
Rejection	Disagreement or disapproval of a proposition or action
Non-existence	Absence of an entity in an expected context
Denial	Negation of the truth of a previous proposition
Prohibition	The act of prohibiting an action or activity
Failure	Negating the successful execution of an action or, unaccomplished goals
Inability	Negation of physical ability
Epistemic negation	Negating possession of knowledge
Other	Other complex categories of negation in surrounding speech

#### Sentence type

In addition to coding for negative functions, the utterances were systematically categorized based on the type of sentence used to fulfill the specific purpose of the utterance. Table 4 indicates the four sentence types used to classify negative utterances.

**Table 4.** Classification of negative utterances with regards to sentence type.

Sentence type	Function	Example
Declarative	Negation of a statement	*We do not have a bag.*
Imperative	Giving a negated demand or instruction	*Don’t move!*
Interrogative	Negation of a question	*Don’t you like it?*
Exclamative	Expressing a strong surprise or emotion with negation	*This is not true!*

Negated declarative utterances convey statements or assertions that express denial, disagreement, or a negative viewpoint. These utterances enable speakers to express their refusal, rejection, or disbelief regarding a certain statement (e.g. *I do not like this*). Negated interrogative statements incorporate negation within a question, such as “*Why didn’t you walk with me?*”. They are used to seek confirmation or clarification while implying a negative assumption or expectation. Negated questions are more complex than declarative utterances because they differ not only in their syntactic structure but also in the type of response they typically elicit (Romero & Han, 2004). Negative utterances manifest as imperatives such as “*Don’t go there!*”, are used to issue commands or requests in prohibitive or negative manner. This linguistic form serves as a clear and direct way to communicate instructions while emphasizing the negated aspect of the command or request. Negative utterances that express strong emotions are coded as exclamative utterances (e.g. *Oh no!*).

#### Data coding

We coded negative functions and sentence types based on their contexts of occurrence in transcribed text of the corpora, as well as audio and video recordings when available. Three trained coders participated in the categorization process. Each coder underwent a training session led by the first author with detailed guidelines on identifying and classifying negative utterances. During this training, the coders reviewed examples of both child speech and child-surrounding speech, and discussed the contextual factors that might influence classification. Additionally, the coders were required to practice coding on a set of sample transcripts, followed by a review session to address any discrepancies and ensure consistency in application of the criteria. When available, the coders also checked the audio and video recordings to ascertain the negative function of the utterances by taking into account factors such as tone, intonation, and body language that could provide additional context for the negative statements. One-word utterances were coded based on the context they appear in. We analyzed the conversational turns between the surrounding speakers and the children, and checked the audio and/or videos to examine the context of one-word utterances to determine their function. Polar questions (e.g. Mother: *Is it raining outside?* Child: *No*) were coded by conducting a detailed analysis of the interactional context. Rather than focusing solely on the polar question and its response (such as the English *no*), we examined the entire conversational turn and the context in which it occurred to inform our decision. In addition to analyzing the conversational context, we examined the non-linguistic context of utterances to accurately interpret the intended function of negation. Non-linguistic context includes physical actions, gestures, facial expressions, and the situational environment surrounding the interaction. By considering these elements, we could more reliably infer what each instance of negation was meant to convey. For example, if a caregiver says “*This doesn’t go in there*” while physically blocking an action or guiding the child’s hand away, it likely functions as a prohibition. In contrast, if the caregiver says the same phrase after the child attempts to place an object incorrectly, it could signify a statement about failure. Inter-rater reliability was assessed using Cohen’s kappa (mean = 92, range = 86–97). This was independent of whether video was accessible in addition to audio (Inter-rater reliability of each file for the language set is given in Table 8 in the Supplemental Materials). To examine the potential impact of CDS on the development of negative functions in child speech, we also categorized negative utterances in the ambient language as either child-directed or child-surrounding.

#### Measures of development

As a measure for the development of negative functions in child speech, we used two metrics applied to each age bin of the children. The first metric measured the ratio of negative functions in all negative utterances produced by children and surrounding speakers. To capture the production of negative functions at a specific age bin while considering the cumulative production of all negative utterances with the same communicative function prior to and following that age bin, we used a sliding-window approach (Henzinger et al., 2009). This approach involved restructuring data with a window size of two by merging sessions of every 2 consecutive months in age, with a monthly interval. The merged data sets were then labeled with the age of the last session. For example, within the age range of 30 to 33 months, three merged data sets were created, representing speech from months 30 to 31, 31 to 32, and 32 to 33, respectively. These datasets were marked as 31, 32, and 33 months in age. To account for variations in the corpus size, the data were bootstrapped 10 times. This method allowed us to capture the production of negative functions within a specific age range while also considering the cumulative production of all negative utterances with the same communicative function, both before and after that age bin. By implementing this standardization method, it became possible to make more reliable comparisons regarding the developmental stage of a negative function at a specific age bin because the recording schemes within our language sample differ, with some recordings conducted on a monthly basis and others on a weekly basis. Furthermore, this approach helped to reduce the impact of noise or short-term fluctuations in the data set and create a more balanced assessment of the overall development leading up to that age bin in time.

#### Statistical modeling

To assess the development of negative functions in children’s productions, we employed mixed-effects regression models using *lme4* (D. Bates et al., 2009) in R (R Core Team, 2022). Our analysis focuses on examining the development of negative functions with the ratio of each negative function by evaluating the ratio of each negative function to the total number of negative utterances within each age bin. The selected model includes the main effects of age, along with random intercepts for negative functions and language. This model, expressed as: ratio ∼ age + (1 | negative function) + (1 | language), was determined to be the optimal choice for representing the data set after model comparisons based on degrees of freedom in ANOVA tests (see Supplemental Materials for all models and their comparisons). We then categorized utterances as either child-directed or child-surrounding for each negative function that children were exposed to in the ambient language in each monthly bin. To evaluate the impact of child-directed input quantity, we included it as a fixed effect in our analysis (tokens ∼ age + child directed + (1 | negative function) + (1 | language) (Please see the OSF repository [https://osf.io/gcfja/] for the data and coding).

## Results

### Study 1: Distribution of negative functions in child and surrounding speech

**Figure 1.**
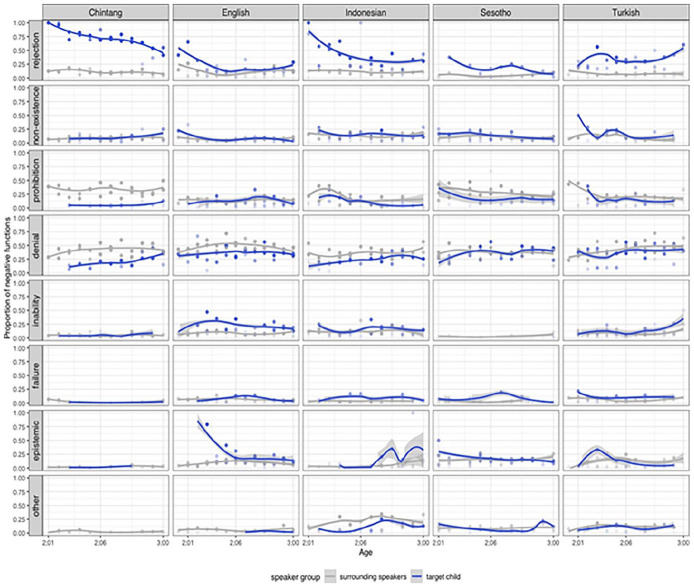
The development of negative functions in child speech relative to surrounding speech. The lines represent smoothed conditional means fitted to the data of two children for each language.

To gain an overview of negative functions in language learning, we first examined the developmental trends for each function in children’s speech compared to child-surrounding speech, including all utterances. Figure 1 illustrates the developmental trajectories of each negative function for target children compared to surrounding speakers across the five languages.

At the onset of age two, *rejection* is by far the most frequent function in Chintang and Indonesian. However, over time except for Turkish, the proportion of *rejection* in child speech decreases. In Turkish, we observe even a slight increase in the use of *rejection* over time, particularly in the last few months across the age span. Notably, Chintang demonstrates the most pronounced decline in the use of *rejection* because almost all negative utterances in child speech are in the form of *rejection* at the onset of age two. *Non-existence* maintains a consistent pattern in child speech across all languages except Turkish, where we observe a declining trend. In all of the five languages examined, children use *prohibition* sporadically, while adults use it consistently throughout the age span. There are notable differences in the use of *prohibition* in adult speech across the languages investigated. Chintang-speaking adults use more prohibitive language compared to adults speaking English, Indonesian, Sesotho, and Turkish. All languages show a slight upward trend in the use of *denial* by children, but in English, the curve is more stable across the age span. The most notable change in the use of *denial* in child speech is particularly evident in Chintang. It seems that children learning Chintang transition from expressing *rejection* to incorporating more *denial* in their negative utterances. Children express *inability* in all languages except in Sesotho, for which we observe only few instances across the age span. The highest use of *inability* is observed in English. Children learning Chintang appear to use it less frequently compared to children learning English, Indonesian, and Turkish. *Failure* has a flat trend in child speech and it does not appear in all age bins. Chintang has the lowest use of *failure* in child speech in comparison to the other languages. While there is a consistent pattern of *epistemic negation* in the ambient language, its usage appears to exhibit considerable variation in child speech across five different languages. While a decreasing trend is observed in English and Sesotho, the use of *epistemic negation* increases over time in Indonesian. Children learning Chintang use *epistemic negation* with very few instances compared with the children learning English, Indonesian, Sesotho, and Turkish languages. The category of *other* negative functions encompasses more sophisticated negative meanings such as inferential negation and appears mostly in adult speech across all languages. Children learning Indonesian, Sesotho, and Turkish use negative utterances with more complex meanings ingrained in the *other* category compared to children learning Chintang and English. Surrounding speakers use more sophisticated negative functions in the other category across all languages. Yet, we observe some differences between the languages, with Indonesian exhibiting the highest proportion in the *other* category (also see Figure 13 in the Supplemental Materials).

**Figure 2.**
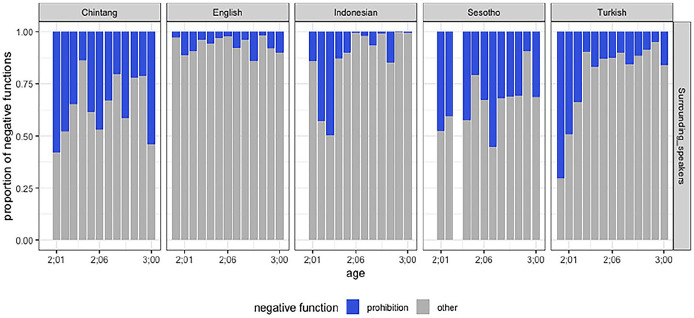
The distribution of prohibition in the ambient language across five languages.

An important finding from the distributions of negative functions is that English-speaking caregivers use *prohibition* less frequently in their input compared to caregivers of other languages (Figure 2). This suggests that English caretakers make use of other linguistic strategies in guiding the behavior of children such as using alternative constructions (e.g. *Stop doing that!*) or expressing guidance through questions (e.g. *Why don’t you . . .?*).

**Figure 3.**
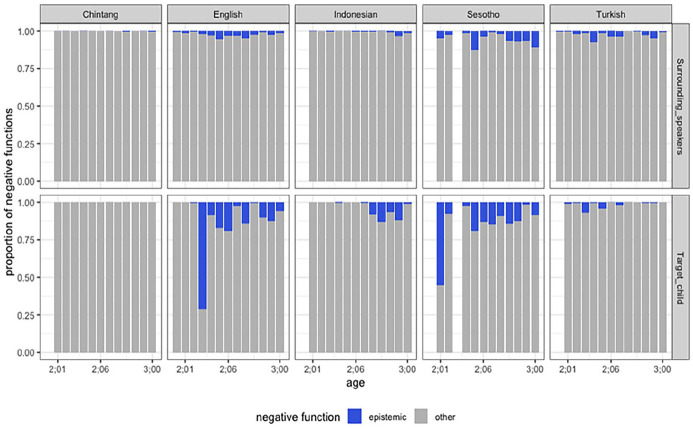
The distribution of epistemic negation in the ambient language and child speech across five languages.

Another interesting pattern is revealed in the use of epistemic negative utterances by children. Epistemic negation is quite rare in child speech compared to other negative meanings (Figure 3). Children learning English and Sesotho express their state of knowledge more than children learning other languages. Children learning Chintang use very few instances of epistemic negation, which reflects the amount of epistemic negation in the input. However, the most important finding is that all functions are used from early on in development with rejection, prohibition, and denial digressing most from adult usage.

### Sentence type and negative functions

**Figure 4.**
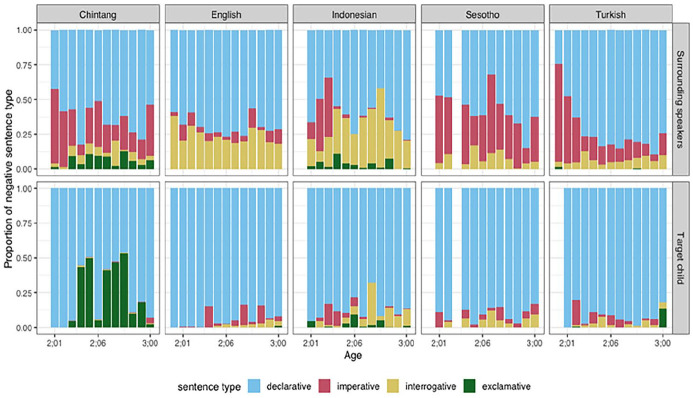
Distribution of sentence type by speaker groups across five languages.

Regarding sentence types, children predominantly use declarative negated sentences, and over time, they begin incorporating imperative utterances into their repertoire (Figure 4). Negated questions emerge towards the end of age two but they remain a minor component of children’s negative utterances. In contrast, adults employ negative sentences across various sentence types, with a higher frequency of usage observed in imperative forms.

Declarative negative utterances represent the most prevalent type of negative utterance produced by children across all languages. Imperative forms typically constitute the second most common category in child speech. Exclamative utterances are rare; however, children learning Chintang produce a higher proportion of exclamative negative utterances. Interrogative forms are more frequently observed in the speech of children learning Indonesian and Sesotho, although they begin to appear in English and Turkish towards the end of the second year of life.

**Example 1:** [Chintang]

***Mother:*** pemsandeP

 ‘*He took out of his mouth*’.

***Child:*** ma-mett-ha! (to his brother)

 NEG-do-NEG.IMP

 ‘*Don’t do!*’

***Child:*** ah mane! (to his mother)

 no

 ‘*Oh no!*’ [CLLDCh1R05S01.1014, 2;05.13]

### Study 2: Development of negative functions

To find out what explains the development of negative functions and whether there are any differences across different language settings, we ran mixed-effects regression models. The selected model tests the main effect of age, along with random intercepts for negative functions and language (ratio ∼ age + [1 | negative function] + [1 | language]).

**Figure 5.**
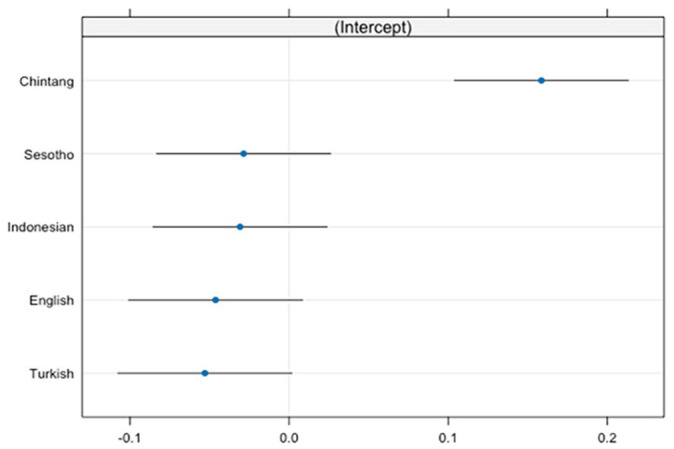
Random intercepts of language.

The results are presented in Table 6 in the Supplemental Materials and Figure 5. The results show a negative effect of age on the development of negative functions (β = −1.39, *t* = −240.93), which implies that over time some negative functions appear less in children’s productions.

**Figure 6.**
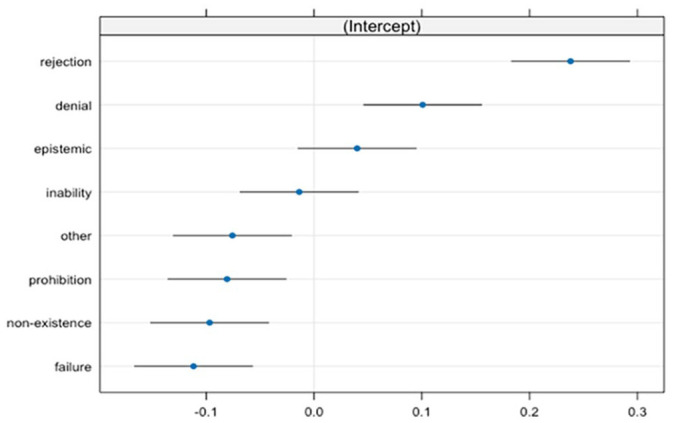
Random intercepts of negative functions. The bars represent 95% confidence intervals.

The results for the language variable as a random intercept suggest variation in the use of diverse negative functions (β = .007, *SD* = .08) across different languages (Figure 5). In our language set, the intercept for Chintang (β = .16) suggests a propensity towards higher proportions compared to the overall model average. While the intercepts for Indonesian (β = −.03) and Sesotho (β = −.03) overlap with the baseline, the intercepts for English (β = −.05), and Turkish (β = −.05) indicate lower proportions, despite the overlap with the baseline (Figure 6).

The intercepts associated with different categories of negative functions reveal distinct patterns. For instance, rejection exhibits a notable positive effect, with β = .23, suggesting higher proportions relative to the overall model intercept. Similarly, denial (β = .10) suggests an increase in proportions together with epistemic negation (β = .03), which leans towards higher proportions, while inability (β = −.01) and failure (β = −.11) suggest a decrease. Conversely, prohibition and non-existence display negative effects compared to the model’s average, with β = −.08 and β = −.09, respectively.

### The effect of child-directed speech

**Figure 7.**
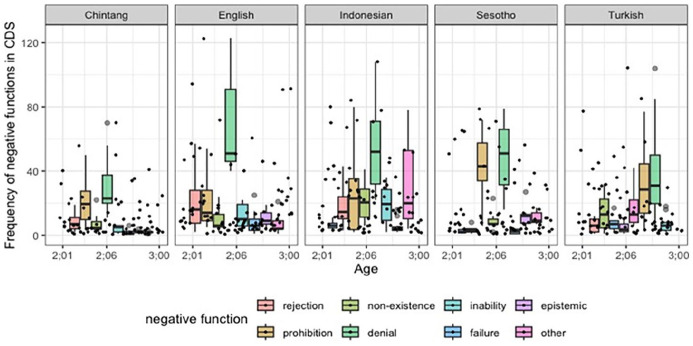
Frequency of negative functions in CDS. The graph summarizes overall number of child-directed negative functions in all negative utterances per age bin for each language. *Note*. CDS = child-directed speech.

Our findings reveal language-specific patterns observed in the distribution of various negative functions in children’s speech, which might be reflecting the influence of the amount of negation in CDS they hear. To explore the potential impact of CDS, we analyzed the frequency of negative functions present in the CDS that children are exposed to in the ambient language. Figure 7 presents an overview of the total number of child-directed negative functions per age bin for each language. In all languages, children encounter a diverse range of negative functions through direct exposure in relatively small quantities. When compared to other languages, Chintang appears to have the lowest frequency of negative functions in CDS. The frequency of *denial* in CDS is higher in English than in the other languages, especially when compared to Chintang. The quantity of direct *prohibitions* that children are exposed to is greater in Sesotho, Turkish, and Chintang compared to other languages.

To investigate the potential effect of CDS on the development of negative functions in children, we examined the use of negative functions in CDS per each age range and then tested its effect on the development of negative functions in a mixed-effects regression model. The results of statistical modeling show a meaningful effect of child-directed input on the development of negative functions (β = 6.54, *t* = 34.52; see Supplemental Materials).

## Discussion

This study offers insights into the development of negative functions across culturally and linguistically diverse contexts. A key finding is the absence of a clear timeline in the development of these functions. Instead, all negative functions are used from early stage across the five linguistically and culturally diverse contexts. The developmental patterns observed in these languages highlight significant cross-cultural variation in the emergence of negative functions. These variations may stem from factors such as differences in social and environmental contexts, specific communicative situations, family interaction styles, and cultural approaches to child-rearing practices (Lieven, 1994; Ochs & Schieffelin, 2011; Stoll, 2016). While many negative functions appear to be accessible to children as early as age two, their frequency and distribution vary across cultural contexts.

Contrary to earlier research suggesting that *denial* develops later in a child’s repertoire of negative expressions (Drozd, 1995; Hummer et al., 1993; Pea, 1980), our findings show that *denial* is used by children as early as age two across all languages. For English-speaking children, *denial* negation has been reported to appear as early as 18 months (Nordmeyer & Frank, 2018). In our language sample, *denial* is frequent and proportionally well represented in English, Sesotho, and Turkish (Figures 13, 16, 20, 22, 27, 30, and 33 in the Supplemental Materials). This indicates that the acquisition of this function is not entirely dependent on the cultural context. Our findings suggest that the use of *denial* increases across all languages (except for English with a constant distribution), while *rejection* shows a decrease (Figure 1). This pattern aligns with previous research, which indicates that *rejection* emerges early in development, while *denial* tends to appear later in child speech (Bloom, 1970; Feiman et al., 2017; Pea, 1980). This exceptional increase in Turkish may be due to the recording contexts, where the higher frequency of *rejection* in the last age bin could potentially be influenced by the specific situations in which the recordings were made and the child used rejections more in the given context. This increase in rejection might indeed be an outlier. The high frequency of *rejection* observed in Chintang-speaking children illustrates the diversity of developmental trajectories. There is also considerable language-specific variation in how often *inability* is expressed in the different languages. Except for Sesotho children, all children express inability from early on. This may be due to linguistic features in Sesotho, where the standard negation marker *ha-* conveys multiple negative meanings, including non-existence (e.g. *ha hona lehlwa* ‘There is no snow’), inability (e.g. *ha ke utlwe* ‘I can’t hear’), epistemic negation (e.g. *ha ke tsebe* ‘I don’t know’), and denial (e.g. *a ha ke a se khaola* ‘No, I did not cut it’). Furthermore, cultural and social norms within the Sesotho-speaking community may further influence the low frequency of specific *inability* expressions. Epistemic negation is more commonly observed in early speech in some languages (such as English and Sesotho) but rarely appears in others (such as Chintang). The rare appearance of epistemic negation in Chintang might be attributed to cultural factors that influence the types of interactions and linguistic input children receive. More fine-grained analyses of these patterns and speculation about potential reasons need to be left to future systematic analyses of contextual and cultural differences.

We observe a lower frequency of *prohibitive* utterances in English compared to other languages. This difference may be attributed to a preference for alternative, non-negated commands (e.g. *Stop touching this!*). As anticipated, prohibitions occur less frequently in children’s speech than in the speech of surrounding adults, likely reflecting the influence of social hierarchy. This function is underpinned by a power dynamic, to which children may become attuned from an early age (Dodane et al., 2014). In many cultures, children are typically positioned at the lower end of family hierarchies making them less inclined to assert authority or impose rules or restrictions on others. Prohibitive language, when used by adults, reflects this power dynamic, as adults are responsible for setting boundaries and maintaining order. Children, on the other hand, tend to express their opposition through personal protests rather than issuing prohibitions. A child might protest against a directive (by saying *I don’t want to!* or *No!*) but is less likely to prohibit others from doing something, as this would require adopting an authoritative stance.

The quantity and type of language input children receive plays a pivotal role in their overall development (e.g. Bergelson et al., 2019; Casillas et al., 2021; Ochs & Schieffelin, 2011). We identified a significant impact of negation in CDS on the development of negative functions in child speech, within our culturally and linguistically diverse language set. Our findings revealed that not all children receive the same quantity of direct negative input in their language-learning contexts and this impacts their own use.

With regard to sentence type, children primarily use negation in declarative sentences, followed by imperative and interrogative sentences. Negative interrogative forms emerge in children’s productions towards the end of age two. For the use of negative questions, there are notable differences observed between adult speech and child speech. This is possibly because negative question forms are syntactically more complex than declarative negative utterances (Romero & Han, 2004). This trajectory is likely due to the relatively low cognitive and syntactic demands of declarative statements (Geffen & Mintz, 2017; Zaitsu et al., 2021), which allow children to express basic forms of negation (e.g. rejecting or labeling non-existence). As they grow, children gain the ability to manage more complex sentence structures, such as imperatives that involve directives or prohibitions (e.g. *Don’t touch that!*) and interrogatives that require processing alternative possibilities or hypothetical scenarios (e.g. *Is it not here?*). The presence of imperatives and questions reflects not only the growing complexity of their negation use but also their expanding communicative intent, where they are able to express more nuanced social intentions (Bellugi, 1965).

Children learning Chintang demonstrate an inclination to use exclamative negative utterances in contrast to children in other languages. In contrast to English, it is observed that surrounding speakers of Chintang, Sesotho, and Turkish exhibit a higher frequency of negative utterances expressed in imperative forms (Figure 4). This notable difference highlights cultural and linguistic practices within these communities, where the use of prohibitive or commanding language seems to be more prevalent. Such linguistic practices serve as a communication tool for expressing restrictions, directives, or adherence to societal norms, and potentially reflect cross-cultural variations in language socialization practices (P. Brown & Gaskins, 2014; Gaskins, 2006).

Our findings support the ample evidence from the literature that language development is characterized by variation (e.g. E. Bates et al., 2017; Hirsh-Pasek et al., 2015). We find substantial cross-linguistic variation in the rate of development of negative functions in the children of our sample of five languages (see Supplemental Materials for individual children). These differences in the rate and course of development between languages are conditioned by a combination of several environmental and maturational factors (E. Bates et al., 2017; Nelson, 1981). The factors underlying this variation – whether pertaining to the quantity and quality of linguistic input, gender differences (Leaper & Smith, 2004), or the specific interactional dynamics within children’s familial environments (Hart et al., 1997) – remains to be explored.

Our results highlight the need for future research to pay closer attention to the recording context. Both the distribution of linguistic forms in the sample and the learning context can vary significantly, particularly with regard to whether interactions are primarily dyadic (one-on-one with a caregiver) or polyadic (involving multiple participants). In dyadic settings, children are likely to receive more direct, tailored feedback, and engage in more structured conversational turns, potentially leading to different patterns of negation use compared to non-dyadic settings, where interactions may be less focused on the child and involve more complex, multi-party exchanges. Future studies with larger, more representative samples across diverse linguistic and cultural contexts would help clarify these patterns and provide a more comprehensive understanding of individual and cross-linguistic variability in the development of negative functions.

Another potential explanation for the differences in the use of negation observed across languages may stem from the complexities inherent in their linguistic structures (Çabuk-Ballı et al., 2025) (see Supplemental Materials for the description of negative functions across five languages). The formal realization of negation is notably intricate and displays considerable variation among different languages, contexts, and even individual speakers (Dahl, 1979; Miestamo, 2006). These differences across languages highlight the varying degrees of complexity in expressing negation, which can influence how easily children learn to understand and produce negative constructions. The presence of multiple forms and contextual requirements in some languages may complicate word learning, as it can lead to increased difficulty in recognizing and using different negative functions, especially in the absence of rich linguistic input and contextual cues. For instance, languages like Sesotho, Turkish, and Chintang with their rich systems of negation present children with a wide array of linguistic features. Such languages demand that children navigate an intricate linguistic landscape and add an additional layer of cognitive processing that children need to master. They require children to not only learn multiple forms but also understand their specific contexts of use. This complexity can further complicate word learning and as a consequence learning of negative functions, as children may struggle to recognize and use denial negation, particularly in instances where the linguistic environment lacks rich contextual cues. In contrast, languages with more straightforward negation systems, like English, may offer children a clearer and more manageable framework for understanding negative constructions. The relative simplicity of using *not*, *-n’t*, and *no* can facilitate quicker recognition and production of negation, thereby reducing the cognitive load on young learners.

In sum, considering diverse environmental and cultural variation accompanied by linguistic diversity, children’s rate of development is potentially influenced by environmental variables as well as the extent of exposure to the language. Some aspects of this variation can be linked to disparities in CDS and considerable variation in grammar and language socialization practices (Bergelson et al., 2019; Casillas et al., 2021; Keller et al., 2006; Ochs & Schieffelin, 1995, 2011; Stoll, 2016).

## Conclusion

In child language acquisition, there is a pressing call for cross-linguistic research, asking for a broader inclusion of linguistic diversity in studies to achieve a more comprehensive and representative understanding of the subject (Kidd & Garcia, 2022; Lieven, 1994; Slobin, 1985; Stoll, 2016). This study contributes to a better understanding of the development of negative functions with a wide array of culturally and linguistically diverse settings. Our findings present cross-linguistic evidence for the development of negative functions and reveal distinct language-specific developmental trends in child speech across the age span of 2 to 3. The variations observed in different linguistic settings suggest that cross-cultural differences in child-rearing practices and the amount of child-directed input may play a role in shaping the language-specific developmental trajectories of negative functions.

The variation in language input across cultures and communities highlights the importance of understanding the role of the diversity of contexts in language acquisition. We need to find ways to track the distribution and characteristics of linguistic input over multiple interactional contexts, developmental time, between families, and across different cultural groups. This will allow for a better understanding of how the human cognitive toolkit for language learning can flexibly adapt to variable circumstances, including situations where CDS is infrequent, produced in large part by other children, or primarily restricted to a small number of activities. It would therefore be important to explore underlying developmental aspects within a socio-cultural and pragmatic approach to tear apart the development of each negative function in children’s developing linguistic repertoire. As for cultural diversity, future research should prioritize expanding corpus studies to include larger and more diverse samples from children across a broader range of languages.

## Supplemental Material

sj-docx-1-fla-10.1177_01427237251336806 – Supplemental material for The road to negation: A comparative study of five typologically and culturally diverse languages Supplemental material, sj-docx-1-fla-10.1177_01427237251336806 for The road to negation: A comparative study of five typologically and culturally diverse languages by Sakine Çabuk-Ballı, Aylin C Küntay, Paul Widmer and Sabine Stoll in First Language
