# Supplementary material for: The road to negation: A comparative study of five typologically and culturally diverse languages
Source: First Lang. 2025 Jun 19;45(5):596–621. doi: 10.1177/01427237251336806 (PMC12435774; doi:10.1177/01427237251336806)
Supplement: sj-docx-1-fla-10.1177_01427237251336806 – Supplemental material for The road to negation: A comparative study of five typologically and culturally diverse languages [file sj-docx-1-fla-10.1177_01427237251336806.docx]

# Supplementary materials

## Functions of negation across five languages

Negation is encoded by means of many different lexical and morphosyntactic forms across the languages spoken around the world (e.g., negative markers, neg- ative words, a combination of negative expressions within a clause) (Dryer, 2013). Basic function of negation is to negate a proposition and flip its truth-functional value (de Swart, 2010). The formal realization of negation, however, is far more complex and exhibits significant variations across different languages, contexts, and even speakers (Dahl, 1979). These variations can make certain types of nega- tion, such as denial or epistemic negation, more challenging to recognize without additional linguistic or contextual information, particularly in early language ac- quisition. Negative utterances are not monolithic; they can carry multiple mean- ings depending on the context, and such subtleties can complicate the process of word learning. Therefore, it is essential to explore how these cross-linguistic dif- ferences may affect a learner’s ability to identify and understand specific forms of negation. In the following section, we provide a concise summary of the neg- ative functions observed in the languages examined in this study, with particular attention to how these variations may impact the recognition of different negation types.

### Chintang

Chintang, a polysynthetic language of Kiranti subgroup of Sino-Tibetan spo- ken in Eastern Nepal, has a negation marking paradigm that distinguishes between past and non-past negation (Paudyal, 2015). Negative meanings are conveyed by a number of affixes (in the form of suffixes and circumfixes) as well as interjec- tions and particles that express different semantic functions (Schikowski, 2013). Standard negation marker (*-n*ɨŋ is used in utterances with the meaning of denial, while *ma(i)- -th* is usually used in imperative prohibitive forms. Non-existence is

expressed by *manchi*ʔ and *manche*ʔ), and non-identity is marked by *maha*ʔ (‘is not good, is not allowed, one shouldn’t’). *Hauk* is a form of negation in Nepali that is used in prohibitive utterances by caretakers speaking to children with the meaning of ‘don’t do that/ stay away from that’. *Aha*ʔ and sometimes *maha*ʔ are used as interjections, mostly for the purpose of rejection by children. Epistemic negation is expressed by the standard negation marker (e.g., *akka ta nisuku*ɨŋ ‘I don’t know that’) or an expression with ‘khoi’ (e.g., *khoi koni* ‘I don’t know’). Below are examples for each function of negation in Chintang.

- - 1. a) ***Mother:*** kok pidahaʔ na

rice give-IMP-1SG.S/P-IMP INSIST

*‘Please give me rice.’*

***Child:*** ahaʔ î

NEG

*‘No.’* [CLLDCh1R01S04.056, 2;06.27]

1. ***Mother:*** rɨŋ na manchi khoi naŋ hokkoiʔɨŋ hook top be.not.there where BUT which-LOC hook *‘Here is no hook, where is the hook?’*

***Child:*** khoi rɨŋta manchi where hook FOC be.not.there

*‘Here is no hook.’* [CLLDCh2R11S04.389, 2;11.2]

1. ***Grandfather:*** hanako likhi mah˜akha

2SG-GEN like no-NMLZ

*‘It is not like yours?’*

***Child:*** mahaʔ ni

be.not EMPH

*‘It is not.’* [CLLDCh1R02S01.1118, 2;02.13]

1. ***Grandfather:*** themma gadi mettiyakti what vehicle make-1/2PL.S/P-IPFV-1/2PL.S/P *‘What to make bus.’*

***Child:*** baje um mamettha lo abhaga

grandfather hmm NEG-do.with-NEG-IMP SURP 1SG.POSS-part-NTVZ

*‘Oh don’t do that grandfather oh my turn.’* [CLLDCh2R11S06.266, 2;11.3]

1. ***Mother:*** to bodabeʔ

DEM.UP cowpea-LOC

*‘Up there, in the beans.’*

***Child:*** thanɨŋ

drop NEG

*‘It does not go down.’* [CLLDCh2R03S02.406, 2;03.2]

1. ***Brother:*** thaba lɨga thaba lo

come.across-IMP go.up-IMP come.across-IMP ok

*‘Come here and climb up.’*

***Child:*** hiŋaanɨŋ

be.able-1SG.S/P-IND.NPST-NEG

*‘I can’t climb up.’*. [CLLDCh1R04S02.0294, 2;04.11]

1. ***Mother:*** kattikhera yakkheŋ thukmacekha lo

when vegetables cook-INF-3NGS.P-MNLZ now

*‘What time will they prepare vegetables for us?’*

***Child:*** khoi manchiʔ

I.don’t.know be.not.there

*‘I do not know, there is not any.’* [CLLDCh1R04S06b.1202, 2;04.11]

1. ***Mother:*** theke lasi makhattase

why return-PURP NEG-go-NEG-PST-PRF-IND.PST

*‘Why don’t you go to take cloth?’*

***Child:*** loʔ imma

ok sleep-1SG.S/P

*‘Okay, I sleep.’* [CLLDCh1R04S03.513, 2;04.12]

### English

English, with relatively less intricate morphosyntactic features, marks nega- tion with two main negators (not and no). Negation markers are used along with a set of word classes to express diverse negative functions like non-existence (e.g., *There is no milk*), prohibition (e.g., *Don’t go there*) and rejection (e.g., *I don’t want soup*). Modality interacts with negative functions and conveys a number of meanings. Inability is expressed by the modal verb *can* (e.g., *I can’t open it.*) as well as the modal-like expression *be able to* (e.g., *She is not able to walk*). In the same way, prohibition is expressed by modal verbs as well as imperative marker

(e.g., *You should not touch this* vs *Don’t touch this*).

- - 1. a) ***Mother:*** Do you wanna mash some potatoes? *[Becky, 2;00.07]*

***Child:*** No.

1. ***Mother:*** That one you’ve got.

***Child:*** No Samson tail.

***Mother:*** Samson hasn’t got a tail in there. *[Gail, 2;00.05]*

1. ***Mother:*** What is it? A hat?

***Child:*** No.

***Mother:*** A bracelet?

***Child:*** No. ***Mother:*** Oh ***Child:*** Armbands.

***Mother:*** Armbands, right. *[Gail, 2;00.05]*

1. ***Mother:*** Does Mummy go in the bubbly one.

***Child:*** Don’t.

***Mother:*** Ok, I’m not allowed to. *[Gail, 2;04.07]*

1. ***Mother:*** I think you need that first, then that.

***Child:*** Can’t fit in.

***Mother:*** Is it too big? *[Gail, 2;02.05]*

1. ***Mother:*** You see if you can find that spider, it’s the other way.

***Child:*** Can’t find it.

***Mother:*** Ok, look more carefully. *[Gail, 2;02.12]*

1. ***Mother:*** What shall I do with it?

***Child:*** I don’t know. *[Gail, 2;03.03]*

1. ***Mother:*** Don’t you think you should be giving it to me in my arm?

***Child:*** That hurt you.

***Mother:*** Yes, it’s very painful. *[Becky, 2;06.05]*

### Indonesian

A language of Malayo-Polynesian family, Indonesian encodes negation in three main forms (tidak/nnggak, jangan, belum). Standard negation is expressed

by the negation marker *tidak*^1^, which is used in utterances expressing denial. Negation marker *belum* is imperfective and expresses undone or unfinished certain activities or states (Kroeger, 2014) (e.g. *belum makan* ‘You haven’t eaten yet’). Negation marker *jangan* signals the imperative mood and is used to mark prohi- bition (e.g. *eh jangan ke tangga* ‘Hey, don’t go to the stairs!’). Non-existence is encoded with the existential predicate *ada* that is preceded by *tidak* and *ng- gak* (e.g. *nggak ada aam* ‘There is no meal’) and seldom by adnominal negation marker *bukan*, which also marks denial (e.g. *ini bukan mainan* ‘This is not a toy’) (Butters, 2021). Epistemic negation is often expressed simply through the nega- tion of the lexeme *tahu* (know) together with the standard negator (e.g. *nggak tahu* ‘I don’t know’).

- - 1. a) ***Child:*** he nggak mau

FILLER NEG want

*‘I don’t want to.’ [Michael, 2;4.25]*

1. ***Child:*** tapi nggak ada bola.

but NEG exist ball

*‘But there is no ball.’ [Michael, 2;4.25]*

1. ***Child:*** Bukain open-VOICE *‘Open it.’*

***Grandmother:*** Nggak boleh dibuka!

NEG may PV-open

*‘Don’t open it!’ [Rizkia, 2;1.7]*

1. ***Child:*** ah Mama nggak bisa

EXCL mommy NEG can

*‘Ah, I can’t.’ [Rizkia, 2;9.19]*

1. ***Mother:*** kenapa emang rodanya

why indeed wheel-POSS

*‘What’s wrong with the wheels?’*

***Child:*** enggak tau

1Negation marker *(e)nggak* is a dialectal (Java) form of standard negation and has extensive allomorphy (*y(e)ndak/kagak)*) in the corpus we analyzed. Both *tidak* and *nggak* are categorized as adverbs (Kridalaksana, 1998) and precede the verb functioning as predicate.

NEG know *[Rizkia, 2;10.4] ‘I don’t know.’*

1. ***Mother:*** ni burung.

this bird

*‘This is a bird.’*

***Child:*** bukan burung

NEG bird

*‘No it’s not a bird.’ [Hizkia, 3;0.1]*

1. ***Child:*** aha nggak muat.

EXC NEG contain

*‘Ugh, it doesn’t fit.’ [Larissa, 2;11.11]*

### Sesotho

Sesotho, a Bantu language primarily spoken in Lesotho, has negative mark- ing with extensive allomorphy as well as tonal marking on subject markers and final syllable of verb stems. Negation interacts with mood, tense, subordination (i.e. dependent moods), agreement that is affected by negation rather than tense (Malete, 2018). The standard negator *ha-* marks several negative meanings such as non-existence (e.g. *ha hona lehlwa* ‘There is no snow’), inability (e.g. *ha keutlwe* ‘I can’t hear’), epistemic (e.g. *ha ketSabe* ‘I don’t know’) and denial (e.g. *a ha keasekhaola* ‘No I did not cut it’). The standard negator also marks rejection together with the negative morpheme *se-*, which is also used in the subjunctive mood (e.g. *ha kebatle dijo* ‘I don’t want food’). Prohibition is usually expressed by the negator *s(*ı*)ka-* that is used in negative imperative forms (e.g. *ska matha* ‘Don’t run’).

- - 1. a) ***Child:*** ee ha kemofe

ij NEG sm1s-t=p_om1-v=give-m=x

*‘No I don’t give her.’* [Litlhare_020400cd, 2;04.0]

1. ***Child:*** ha hona metsi ka mona

ng sm17-t=p_v=be-m=x water(6irg) pr loc

*‘There is no water in here.’* [Litlhare_021000cd, 2;10.0]

1. ***Child:*** skaetsa tjena

ng-v=do-m=x av

*‘Don’t do this.’* [Hlobohang_020100ab, 2;01.0]

1. ***Child:*** ha keutlwe

ng sm1s-t=p_v=hear-m=x

*‘I can’t hear.’* [Hlobohang_020200ab, 2;02.0]

1. ***Child:***ha ketSabe

ng sm1s-t=p_v=fear-m=x

*‘I don’t know.’* [Hlobohang_020800ab, 2;08.0]

1. ***Child:*** a ha keasekhaola

ij ng sm1s-t=p-om7-v=cut-m=x

*‘No I did not cut it.’* [Hlobohang_021100ab, 2;11.0]

1. ***Child:*** ha eye ka sakeng.

*‘It doesn’t go in the kraal.’* [Litlhare_021000cd_935, 2;10.0]

### Turkish

In Turkish, an Altaic language, negation marking is mainly encoded with *- mA* that is the primary means used for negating verbal sentences and subordinate clauses. This standard negation marker interacts with tense, aspect and mood. It is used in imperative mood to encode prohibitive utterances (e.g. Gitme oraya ‘Don’t go there’). It expresses all negative functions except for non-existence. Non-existence is marked with *yok*, which is a lexical antonym of the verb exist *var* (*Oyuncak yok* ‘There is no toy’). Non-identity marking *deg˘il* can express denial (e.g. *Dog˘ru deg˘il* ‘It is not true’) in addition to the standard negator. Epistemic negation is marked with the verb *bil-* that is conjugated with the standard negation marker (e.g. *Bilmiyorum* ‘I don’t know’).

- - 1. a) ***Child:*** Ben pasta yemiycem.

I cake eat-NEG-FUT-1SG

*‘I won’t eat cake.’*. [Irem, 2;8.2]

1. ***Child:*** Pis.

*‘Dirty’*

***Mother:*** Peçete yok

*‘There is no napkin.’* [Irem, 2;6.22]

1. ***Mother:*** Gitme oraya. go-NEG there-DAT *‘Don’t go there.’*

***Child:*** Gitme.

go-NEG

*‘Don’t go.’* [Senem, 2;6.26]

1. ***Child:*** Açamıyorum.

I open-ABIL-NEG-PROG-1SG

*‘I can’t open it.’* [Senem, 2;11.24]

1. ***Child:*** Bilmem.

know-NEG-1SG

*’I don’t know.’* [Irem, 2;7.21]

1. ***Child:*** Ben çi(z)medim.

I draw-NEG-PST-1SG

*‘I did not draw it.’* [Irem, 2;9.27]

1. ***Child:*** Çıkmıyo(r) ama.

come.out-NEG-AOR-3SG but

*‘It doesn’t come out.’* [Senem, 2;8.23]

In summary, the linguistic diversity in expressing negation emphasizes the necessity of considering not only the grammatical structures involved but also the contextual factors that influence language learning. As children interact with their linguistic environment, the complexities and variations in negation can either facilitate or hinder their ability to understand and produce negative constructions, based on the richness of the linguistic input they receive and the contextual cues available to them.

## Differences between children and across languages

Children exhibit diverse developmental trajectories in acquiring negative func- tions. The course of acquisition might be influenced by linguistic input, social interaction, and cognitive maturation. Importantly, negation is not solely con- veyed through explicit linguistic markers but also through multimodal means such as gestures, facial expressions, prosodic cues, and alternative syntactic structures (Morgenstern et al., 2016; Dodane et al., 2014; Prieto & Espinal, 2020). In figure 8, we see the similarity in the extensive use of *rejection* by both children learning Chintang across the age span. The frequency of *rejection* as a negative func- tion decreases over time, ranging from 100% to 40-60% of negative utterances.

There is an upward trend in the use of *denial* in child speech over time. Chil- dren rarely use *epistemic* negation and expressions of *failure*, despite the pres- ence of these negative meanings in the ambient language. This tendency may be attributed to the multimodal nature of communication, in which negative ex- pressions—particularly epistemic negation—are often conveyed through gestures or other nonverbal cues rather than explicit linguistic markers. The use of *non- existence* ranges between 10% to 40% across the age span and it is consistently used by the two children, while *inability* appears sporadically across the age span. Children do not use more complex forms of negation that are classified in the *other* category. Both children seem to receive a similar variety of negative meanings in their surrounding speech, with a dominance of *denial* and *prohibition*. The neg- ative concepts of *non-existence* and *rejection* constitute a substantial proportion, ranging from 20% to 40%, of the negative utterances present in the input. How- ever, other negative meanings appear rarely in the ambient speech, which reflects cultural setting of the community.


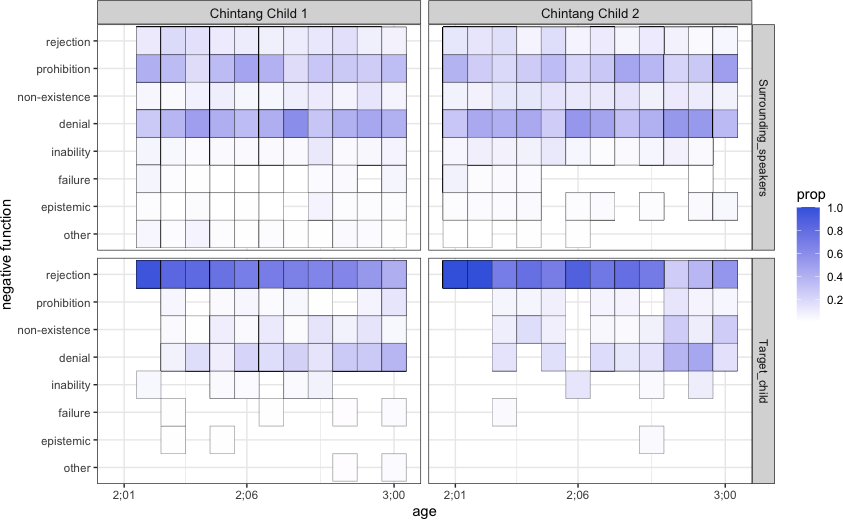


Figure 8: Distribution of negative functions across speaker groups for each child in Chin- tang

As seen in figure 9, children learning English actively use all different neg- ative meanings more frequently compared to children learning Chintang. In line with the input, their use of denial is very frequent, ranging from 20% to 60% of negative utterances. Rejection is the second most frequent negative meaning in child speech. It is used more frequently at the beginning of age two and decreases over time. Child 2 uses epistemic negation (ranging from 20% to 70% across the age span) more frequently than child 1 (ranging from 10% to 50%). Inability and non-existence seem to be distributed similarly across the age span, accounting for

20% to 40% of negative utterances. In the surrounding speech, most of the neg- ative utterances contain denial (ranging between 50% and 70%. Other negative meanings are more or less evenly distributed across the age span. English speak- ing caregivers use different negative meanings compared to Chintang speaking caregivers.


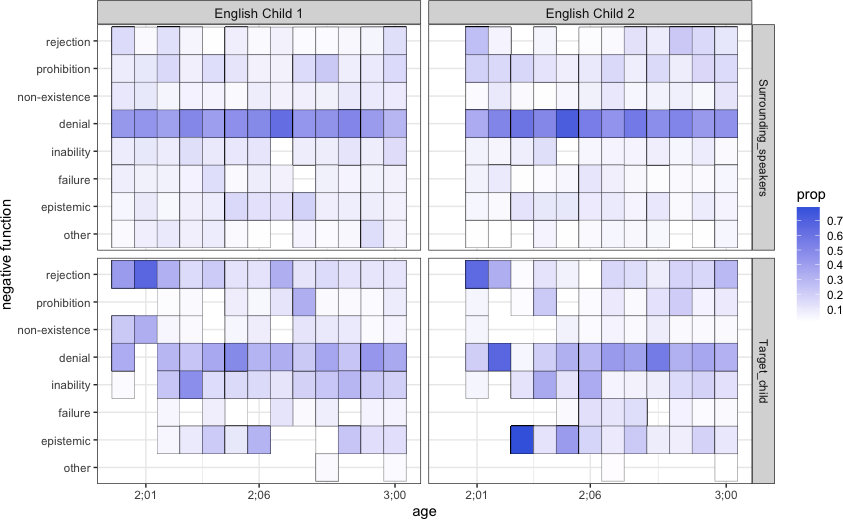


Figure 9: Distribution of negative functions across speaker groups for each child in En- glish

The two children learning Indonesian primarily use negation to express rejec- tion, whereas the surrounding speakers rely more heavily on denial as the primary negative function in their speech. Child 2 exhibits a rising trend in the use of denial, whereas child 1’s use of denial fluctuates. Child 2 appears to be more ver- satile in using different negative functions throughout the age span, while child 1 uses various negative meanings with less diversity. Children learning Indone- sian use more diverse negative meanings across the age span compared to children learning Chintang. The surrounding speakers of both children use diverse nega- tive functions, with prohibition and denial being the most frequently used negative meanings (ranging between 20% and 60% of negative utterances) in the ambient language.

In Sesotho, children predominantly use negation to reject and deny, similar to children learning English. Adults use negation in the form of prohibition and denial. Other forms of negation appear rarely in both adult and child speech. Child 1 appears to use different negative functions more diversely than child 2. Notably, inability does not appear in the utterances of child 2 and child 1 uses it only in a few instances (around 10% of negative utterances). Epistemic negation is relatively frequent in both children’s speech and the ambient language like in


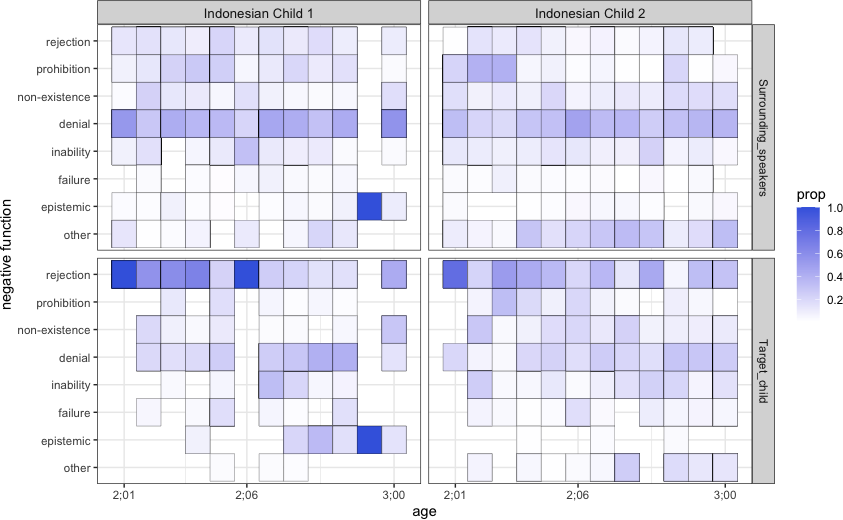


Figure 10: Distribution of negative functions across speaker groups for each child in Indonesian

English.


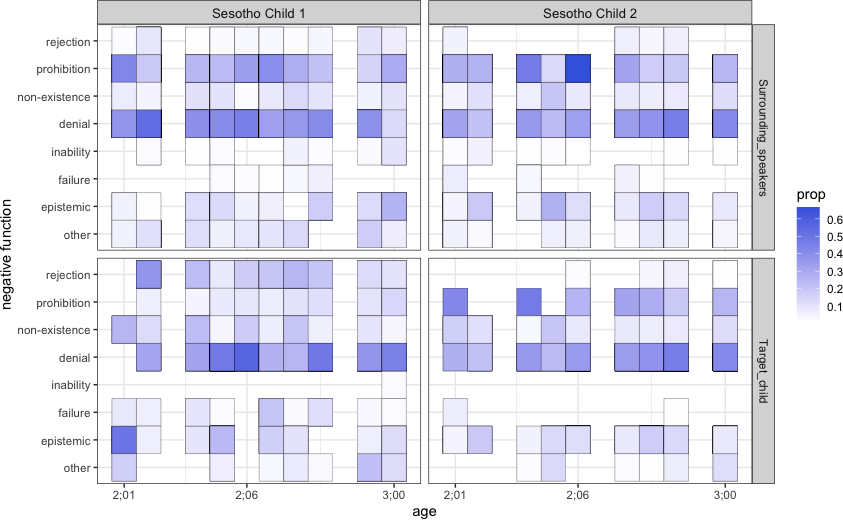


Figure 11: Distribution of negative functions across speaker groups for each child in Sesotho

Turkish-speaking children use negation to express denial and rejection similar to Chintang, Indonesian, and Sesotho-speaking children (ranging between 30% and 60%). Rejection is extensively used (ranging between 30% and 70%) by both children in all age bins. Non-existence is also present in their repertoire, with child 1 using it actively across all age groups and child 2 rarely using it. Child 2 actively uses denial across the age span, while child 1 uses it less frequently,

but with a gradual increase over time. Inability is used more often by child 2 compared to child 1, and all other negative meanings are part of both children’s linguistic repertoire. Denial is the most frequently used negative meaning among surrounding speakers. Adults surrounding child 1 use prohibition more frequently than those surrounding child 2, and accordingly, child 1 uses prohibitive negative utterances more often than child 2.


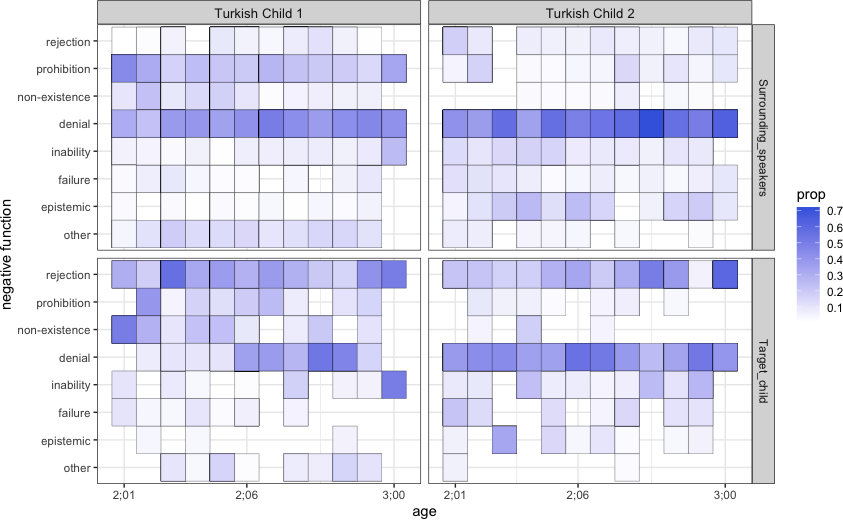


Figure 12: Distribution of negative functions across speaker groups for each child in Turkish

## Strategies to support linguistic expression

Caregivers employ several strategies to support children’s expression of negation. A noteworthy strategy is the reformulation of what the child is trying to express in full grammatical form. Caregivers usually correct or complement children’s utterances by repeating what they have said. This helps the child to hear full linguistic form in the input. It also acts as a form of confirming what the child says. Below are some examples, in which caregivers repeat what is said in a full form and ask the child to repeat an utterance after them.

**Example 1:** [English]

***Mother:*** Do you want your toys?

***Child:*** No!

***Mother:*** Ok, you don’t want your toys.

**Example 2:** [Turkish]

***Mother:*** Uyku zamanı.

*‘It is time to sleep.’*

***Child:*** Uyma [uyuma]

*‘No (no sleeping).’*

***Mother:*** Anladım uyumak istemiyorum diyorsun.

*‘I see you say you do not want to sleep.’*

Another important strategy used by caregivers is asking children to repeat an utterance after them. This strategy by the caregivers is usually referred to as scaffolding. Scaffolding plays an important role in the linguistic development of children and usually involves parents’ verbal input during interactions with their children (Mermelshtine, 2017). In the following examples, scaffolding sets the ground for children to express different negative functions.

**Example 3:** [Indonesian]

***Mother:*** Nggak Tante jangan gitu dong

*‘Say no, Auntie, don’t.*

***Child:*** Jangan jangan

*‘Don’t, don’t.’*

**Example 4:** [Sesotho]

***Mother:*** Ake ujwetse Mami ubatla tee.

*’Please tell Mami you want some tea.’*

***Child:*** Nna kebatla tee yaka.

*‘I want my tea.’*

**Example 5:** [Chintang]

***Mother:*** pempak aã coha ɨo

*’Eat the roti.’*

***Brother:*** maneî mo ludaî na

*’Say no.’*

***Child:*** ahaʔ.

*‘No.*’

Imitation plays an important role in the acquisition of new structures and cod- ing their meanings (Bloom, Hood, & Lightbown, 1974). Children practice neg- ative utterances they hear by repeating or imitating, which greatly contributes to

their linguistic development. Children generally appear to imitate as they process linguistic and nonlinguistic input in the environment (Ex. 6).

**Example 6:** [Turkish]

***Mother:*** Sabun yenmez.

*‘Soap is not edible.’*

***Child:*** Sabun yenmeç(z). (repeating what her mother says)

*‘Soap is not edible.’*

Further, the way children are raised in different cultures and the input patterns influence the development of different negative functions. In western cultures children are exposed to explicit instruction, usually by the mother. As a result, children learn these negative functions more quickly (Ex. 7).

**Example 7:** [English]

***Mother:*** Can you tell me what color the dress is Becky?

***Child:*** Yellow.

***Mother:*** No, it’s not yellow.

***Child:*** Green.

***Mother:*** No, it’s blue, isn’t it?

The mother tries to elicit the correct answer from the child and guides the child to the accurate answer. In the example below, the mother explains that the object at the center of attention of the child is not a ball and guides her to name the object.

**Example 8:** [English]

***Child:*** A ball.

***Mother:*** No, it’s an orange.

***Child:*** A ball.

***Mother:*** It’s not a ball. It’s an orange.

***Child:*** Ball.

***Mother:*** No, it’s not a ball. It looks like a ball but it’s actually an orange.

The examples above show that caregivers use several different strategies to support the development of different negative meanings. Diversity in child-rearing practices and strategies lead to different routes in the development of negative meanings in child speech.

## Results of statistical models

In the statistical models, we test the proportion of each negative function in overall negative utterances per age bin as the outcome variable. We look at the contribu- tion of age, input type as predictors that can explain the development of negative functions in child speech. We look at the differences across languages and nega- tive functions with random intercepts. Predictors used in the statistical models are as follows:

**Proportion (prop)**: The ratio of negative functions relative to overall negative utterances children produce within the age range of 2;00 to 3;00. This ratio is computed based on the number of negated tokens per age bin.

**Negative function**: Negative functions encompass eight negative meanings attested in child speech and the ambient language. These include rejection, non- existence, prohibition, denial, inability, failure, epistemic and the other category (i.e. more complex negative functions such as inferential negative meaning used by surrounding speakers). We introduce negative functions into the modeling ap- proach by incorporating random intercepts. This enables us to investigate varia- tions in connection with these specific categories.

**Language**: The language variable has five levels and is added to the regression models with random intercepts to find out differences across the languages.

**Input type**: The input type is characterized by the classification of input into two categories: negative utterances categorized as child-directed speech and child- surrounding speech.

### Model comparisons

To determine the best fitting model for the data, we conducted ANOVA tests and compared models based on their degrees of freedom, AIC (Akaike Informa- tion Criterion), and BIC (Bayesian Information Criterion) values. These statisti- cal measures helped us assess and select the model that provides the most optimal balance between goodness of fit and complexity.

- - 1. prop ∼ age in months + negative function + (1|language)
    2. prop ∼ age in months + negative function + (1|language) + (1 |target child)
    3. prop ∼ age in months + (1|negative function) + (1|language)
    4. prop ∼ age in months + (1 + negative function|language) + (1 + negative function|target child) (This model overfits because the random effects structure is too complex to be supported by the data)
    5. prop ∼ age in months + (1|negative function) + (1|language) + (1|target

child)

Results of ANOVA for the comparisons:

Table 5: Model Comparisons with ANOVA

| Model | Parameters | AIC | BIC | LogLik | Deviance | Chisq | Df | Pr(*>*Chisq) |
| --- | --- | --- | --- | --- | --- | --- | --- | --- |
| m3 | 5 | -502827 | -502771 | 251419 | -502837 |  |  |  |
| m5 | 6 | -528765 | -528697 | 264389 | -528777 | 25940 | 1 | *<* 2 × 10^−16^ |
| m1 | 11 | -502894 | -502770 | 251458 | -502916 | 0 | 5 | 1 |
| m2 | 12 | -528832 | -528696 | 264428 | -528856 | 25940 | 1 | *<* 2 × 10^−16^ |

Table 6: Results of the regression model with fixed effects

|  | Statistic | Value |  |
| --- | --- | --- | --- |
|  | AIC | -502827.2 |  |
|  | BIC | -502770.6 |  |
|  | LogLik | 251418.6 |  |
|  | Deviance | -502837.2 |  |
|  | df.resid | 610475 |  |
|  |  |  |  |

Scaled Residuals Min 1Q Median 3Q Max

-3.05 -0.63 0.09 0.61 5.02

|  |  | |  |  |  |  |
| --- | --- | --- | --- | --- | --- | --- |
|  | Random Effects | | Variance | Std. Dev. |  |  |
|  | neg_function (Intercept) | | 0.01 | 0.11 |  |  |
|  | language (Intercept) | | 0.007 | 0.084 |  |  |
|  | Residual | | 0.02 | 0.16 |  |  |
|  |  | |  |  |  |  |
| Fixed Effects | | Estimate | Std. Error | t value | |  |
| (Intercept) | | 6.72 | 5.59 | 12.01 | |  |
| age_in_months | | -1.39 | 5.77 | -240.93 | |  |

**Results of statistical modeling about child-directedness in negation**

## Data coding: Cohen’s Kappa

Inter-rater reliability of each file for the language set are given in Table 8.

Table 7: Results of modeling with child-directedness in negation

| **Parameter** | **Estimate** | **Std. Error** | **t-value** |
| --- | --- | --- | --- |
| Intercept | 308.40 | 41.47 | 7.44 |
| Age in months | -6.21 | 0.17 | -37.25 |
| Child-directed | 6.55 | 1.45 | 4.52 |

| **Model Statistics** | **Value** |
| --- | --- |
| AIC | 126748.5 |
| BIC | 126792.6 |
| Log-likelihood | -63368.2 |
| Deviance | 126736.5 |
| Number of observations | 11461 |
| Number of groups (neg_function) | 8 |
| Number of groups (language) | 5 |

| **Random Effects** | **Variance** | **Std. Dev.** |
| --- | --- | --- |
| Neg_function (Intercept) | 13305.2 | 115.35 |
| Language (Intercept) | 141.2 | 11.88 |
| Residual | 3688.0 | 60.73 |

Table 8: Results of inter-rater reliability for each child: Cohen’s kappa

| **Language** | **Children** | **N(Utterances)** | **Cohen’s kappa** | |
| --- | --- | --- | --- | --- |
|  |  |  | **Child** | **Surrounding speakers** |
| Chintang | Child 1  Child 2 | 3696  2178 | 0.84  0.90 | 0.88  0.94 |
| English | Child 1  Child 2 | 1761  2009 | 0.92  0.96 | 0.96  0.98 |
| Indonesian | Child 1  Child 2 | 2283  2074 | 0.90  0.88 | 0.94  0.92 |
| Sesotho | Child 1  Child 2 | 962  1217 | 0.87  0.93 | 0.91  0.95 |
| Turkish | Child 1  Child 2 | 2027  869 | 0.94  0.93 | 0.96  0.95 |

## Proportion and absolute frequency of negative functions in child speech and ambient language

The proportion of negative functions differs across the languages. While sur- rounding speakers demonstrate a broad4e7r range of sophisticated negative utter-

ances, target children typically exhibit a more limited repertoire, focusing ini- tially on simpler forms of negation. At the onset of age two, *rejection* is the most prevalent negative function in Chintang and Indonesian; however, the pro- portion of *rejection* in children’s speech decreases over time in all languages ex- cept Turkish, where it slightly increases. Chintang shows the steepest decline, with *rejectio*n dominating child speech initially. *Non-existence* remains consis- tent in child speech across most languages, except for Turkish, which exhibits a decline. Children sporadically use *prohibition*, while surrounding speakers em- ploy it consistently. Although children show an upward trend in *denial*, English maintains a stable curve, with a notable transition in Chintang from *rejection* to *denial*. *Inability* is frequently expressed by children in all languages except Sesotho, with English showing the highest use. Children learning Chintang use it less than those learning English, Indonesian, and Turkish. *Failure* is infrequent in child speech, particularly in Chintang, which has the lowest instances compared to other languages. *Epistemic negation* varies significantly among languages; while it decreases in English and Sesotho, it increases in Indonesian. Children learning Chintang use *epistemic negation* sparingly compared to their peers. Finally, the *other* category, which includes more sophisticated negative meanings, is predom- inantly represented in adult speech. Children learning Indonesian, Sesotho, and Turkish employ more complex negative utterances than those learning Chintang and English, with surrounding speakers demonstrating a higher proficiency in so- phisticated negative functions across all languages, particularly in Indonesian.


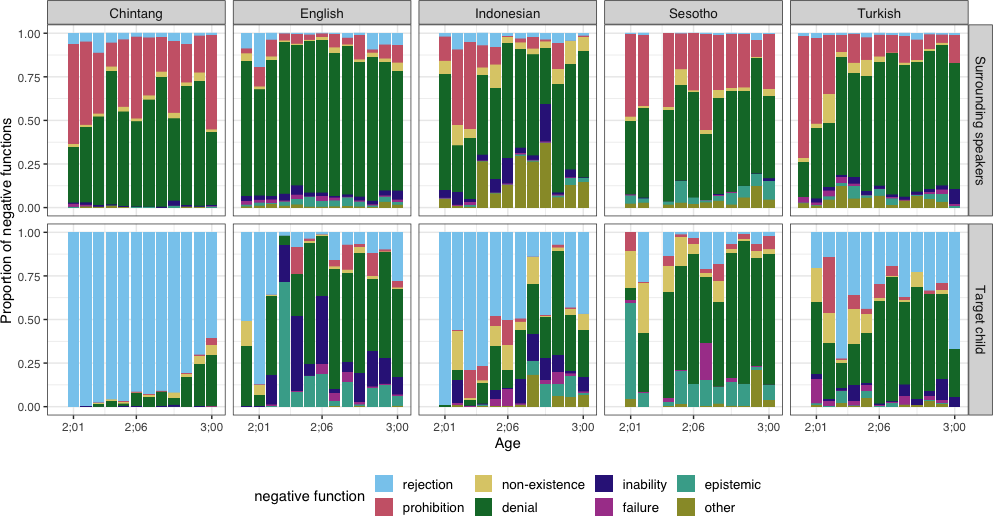


Figure 13: The proportion of negative functions in child speech relative to surrounding speech in five languages


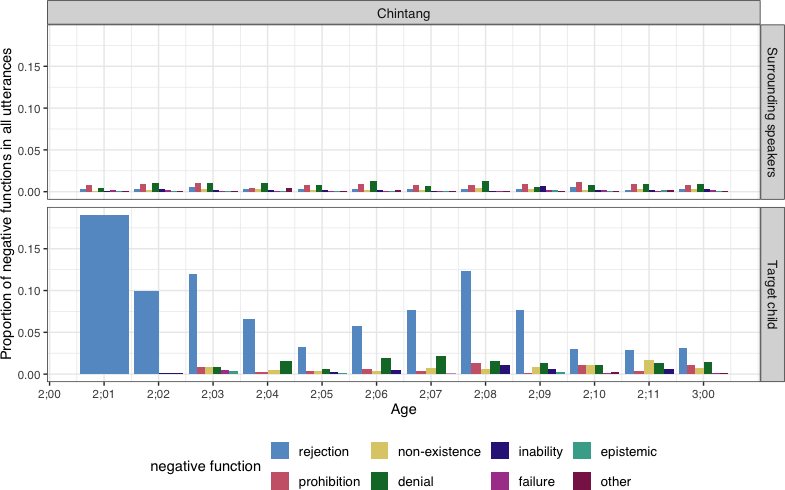


Figure 14: Chintang: The proportion of negative functions in all utterances in child speech relative to surrounding speech


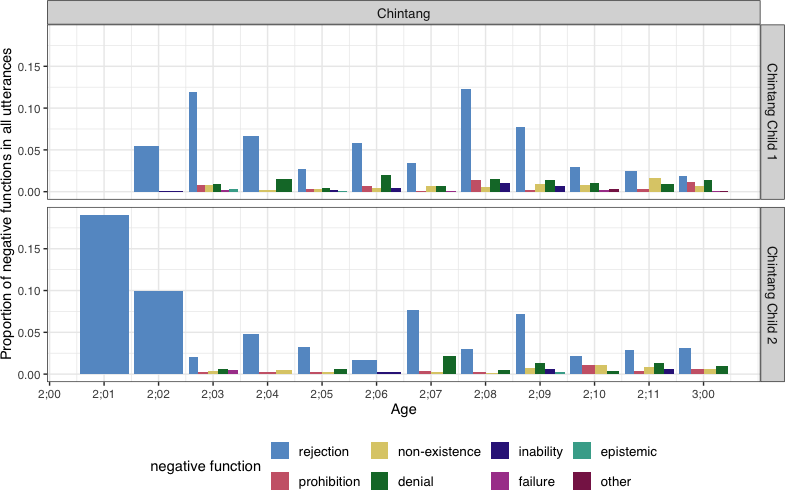


Figure 15: Chintang: The proportion of negative functions in all utterances in child speech


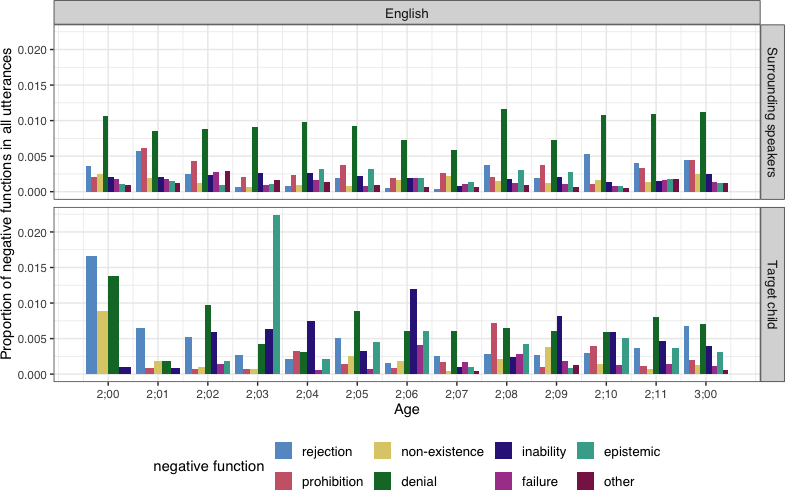


Figure 16: English: The proportion of negative functions in all utterances in child speech relative to surrounding speech


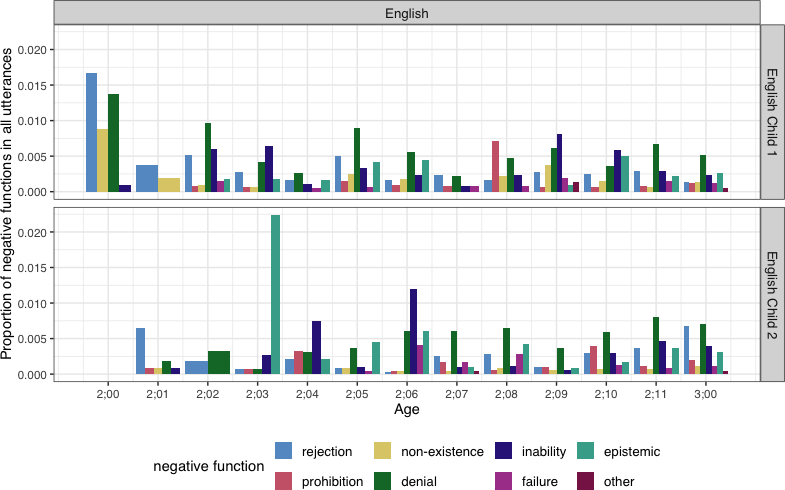


Figure 17: English: The proportion of negative functions in all utterances in child speech


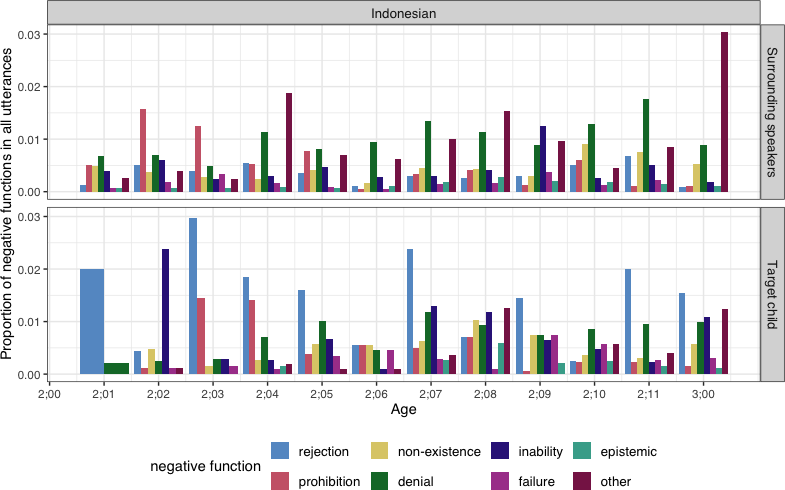


Figure 18: Indonesian: The proportion of negative functions in all utterances in child speech relative to surrounding speech


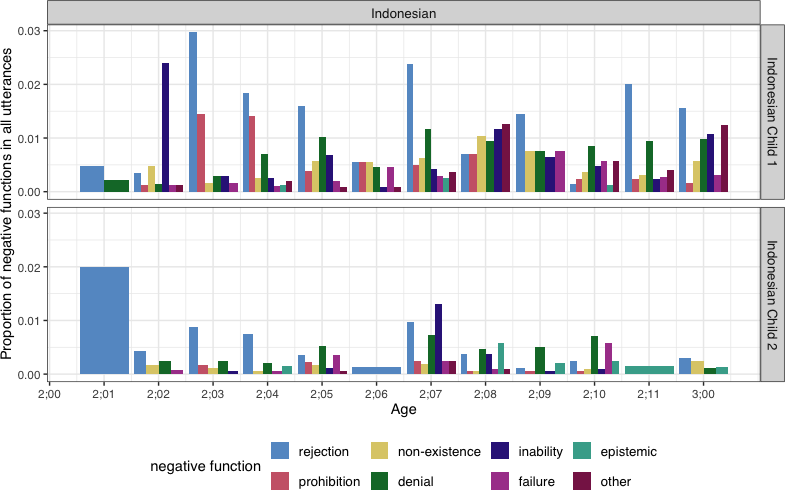


Figure 19: Indonesian: The proportion of negative functions in all utterances in child speech


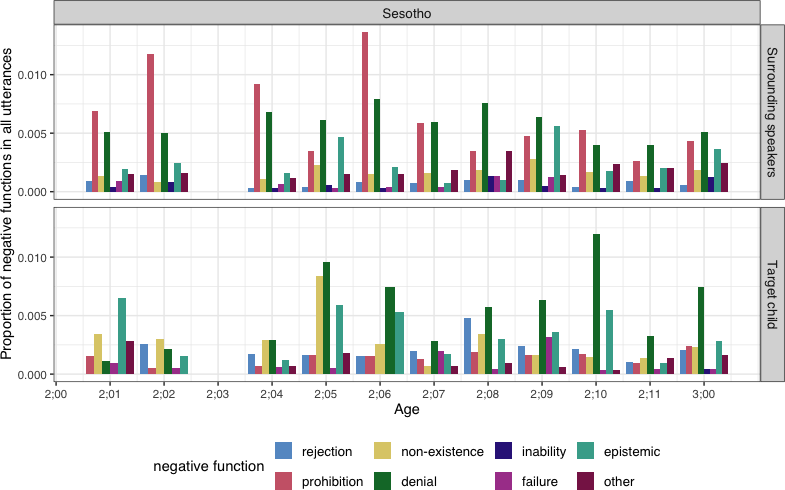


Figure 20: Sesotho: The proportion of negative functions in all utterances in child speech relative to surrounding speech


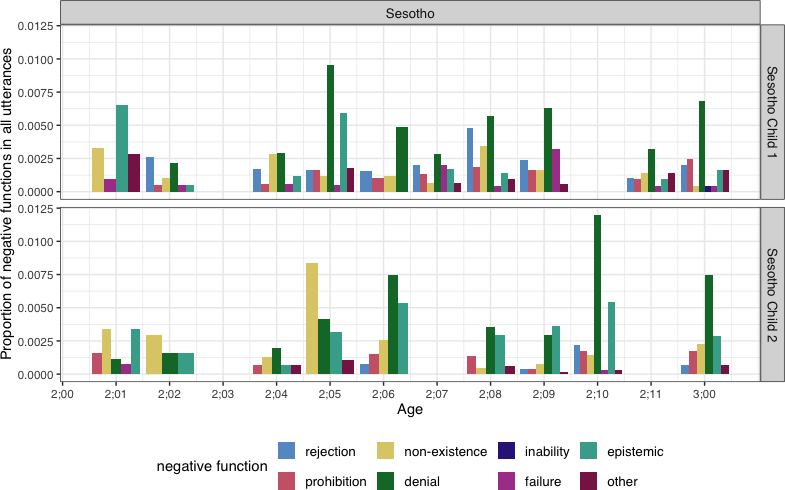


Figure 21: Sesotho: The proportion of negative functions in all utterances in child speech


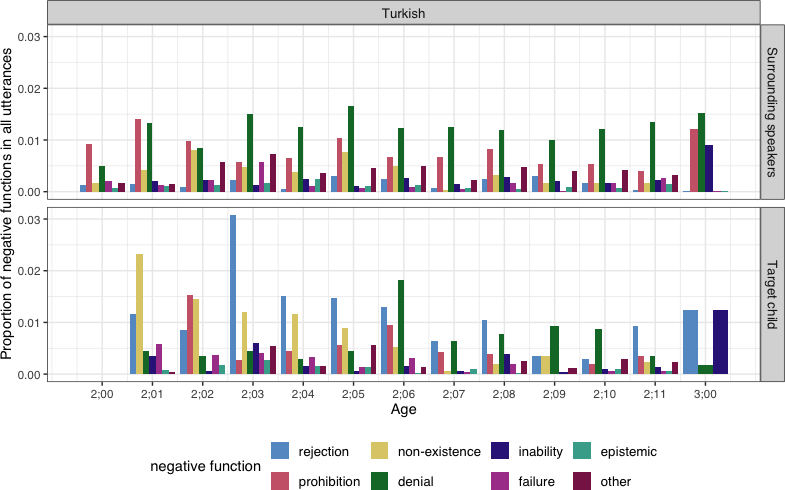


Figure 22: Turkish: The proportion of negative functions in all utterances in child speech relative to surrounding speech


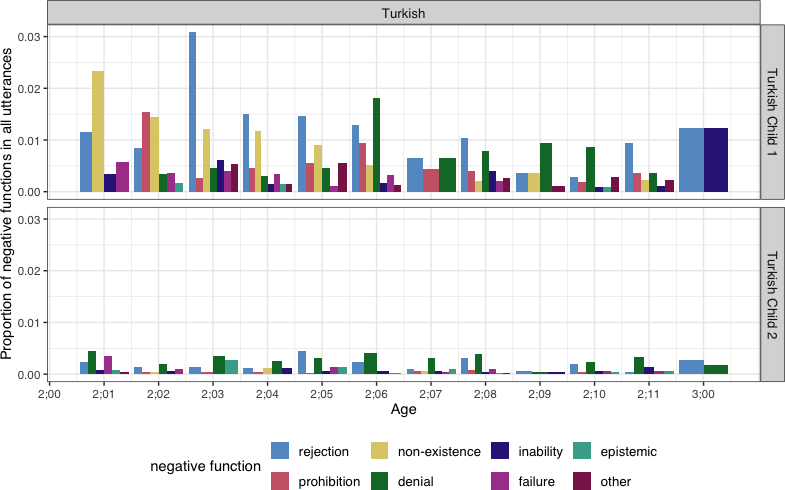


Figure 23: Turkish: The proportion of negative functions in all utterances in child speech


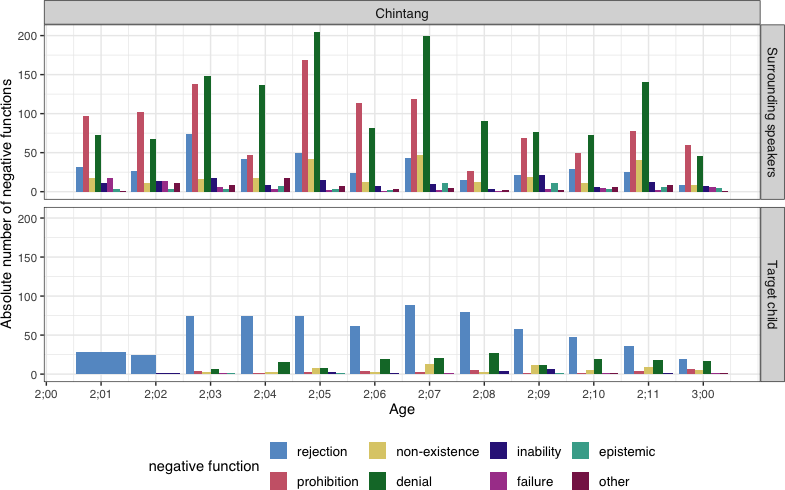


Figure 24: Chintang: The frequency of negative functions in child speech relative to surrounding speech


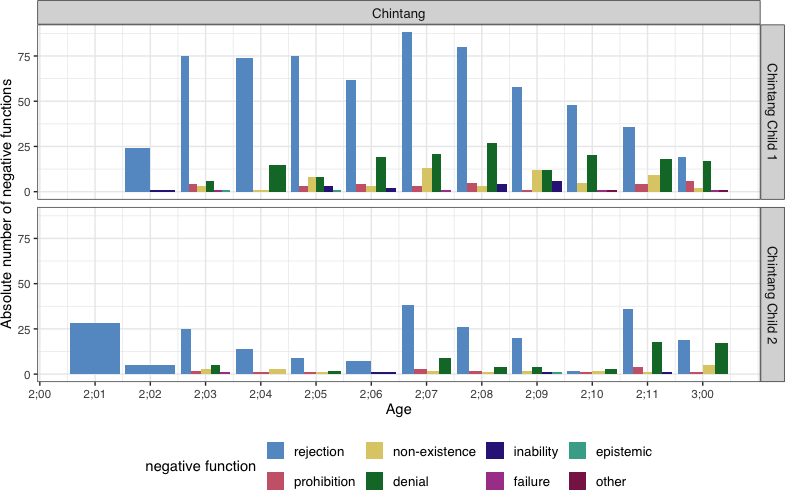


Figure 25: Chintang: The frequency of negative functions in child speech


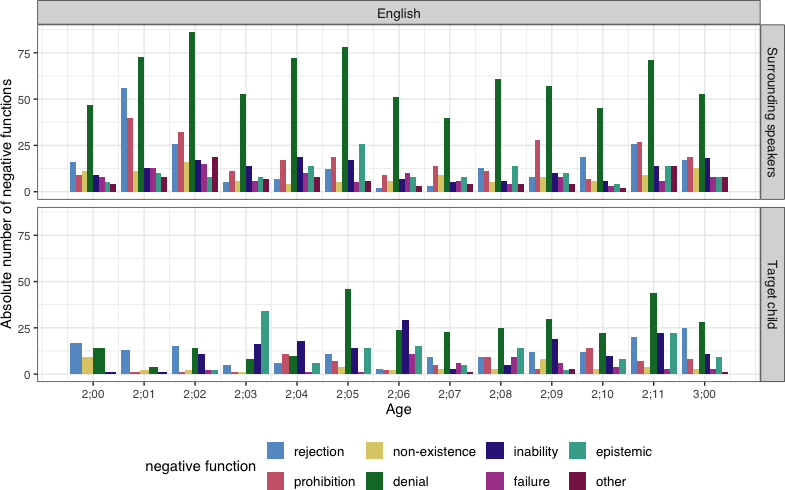


Figure 26: English: The frequency of negative functions in child speech relative to sur- rounding speech


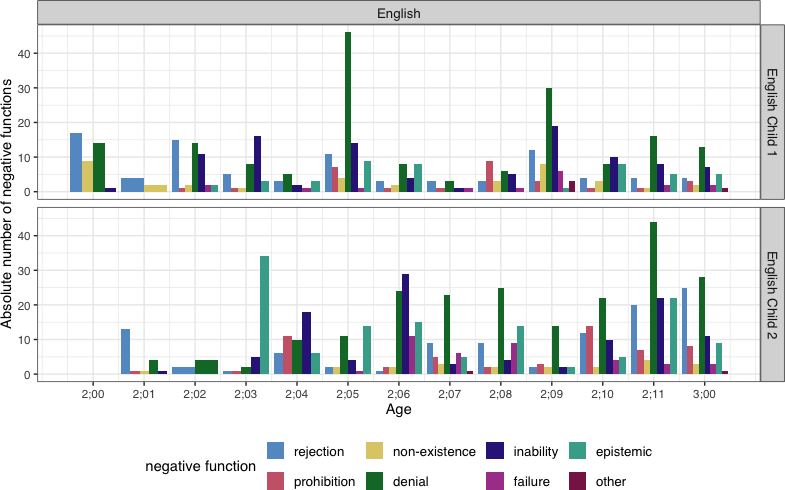


Figure 27: English: The frequency of negative functions in child speech


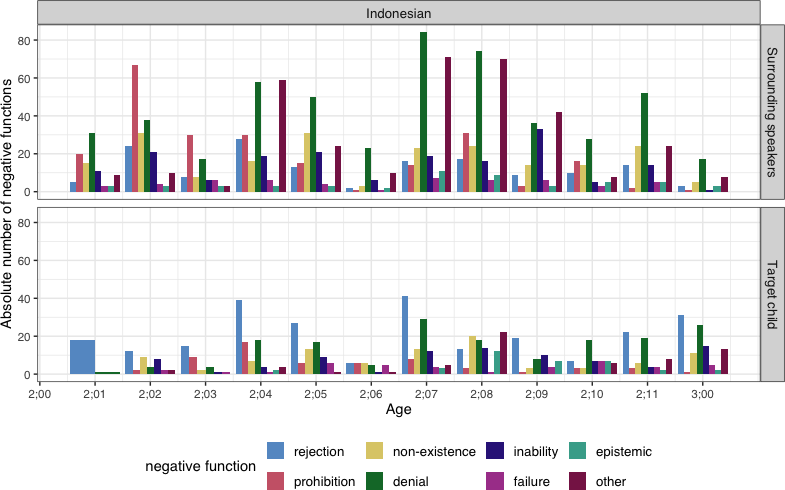


Figure 28: Indonesian: The frequency of negative functions in child speech relative to surrounding speech


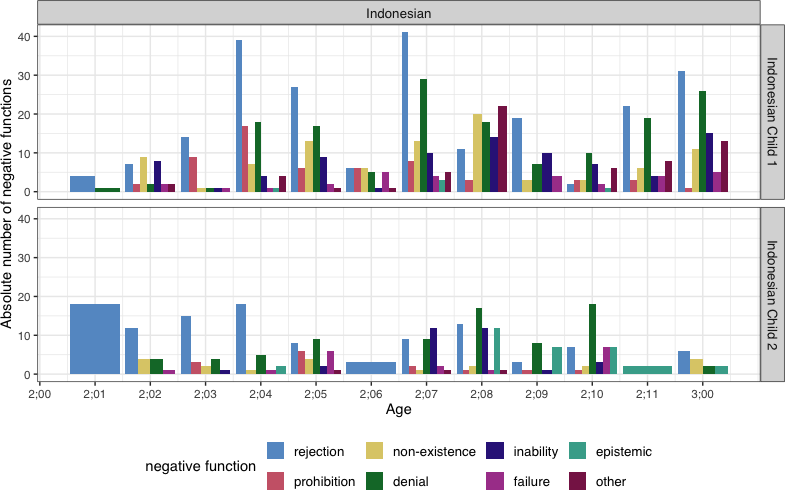


Figure 29: Indonesian: The frequency of negative functions in child speech


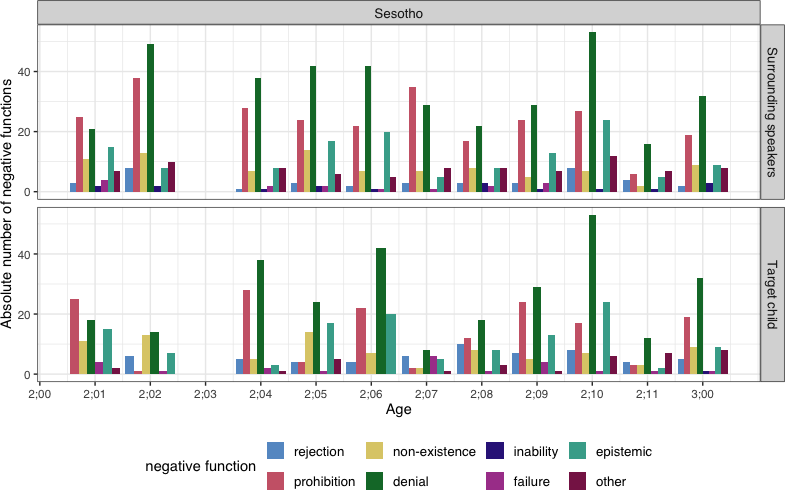


Figure 30: Sesotho: The frequency of negative functions in child speech relative to surrounding speech


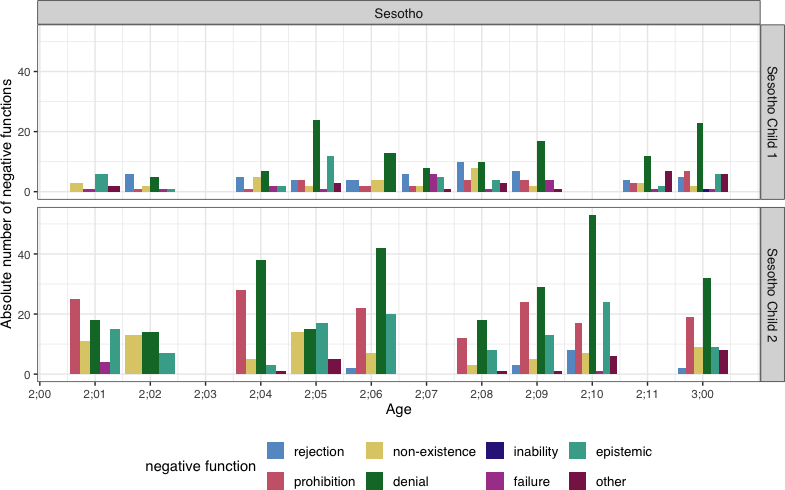


Figure 31: Sesotho: The frequency of negative functions in child speech


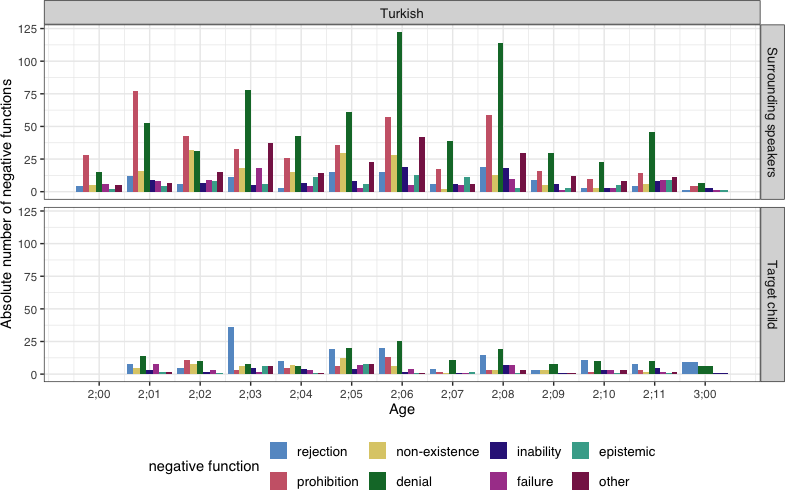


Figure 32: Turkish: The frequency of negative functions in child speech relative to sur- rounding speech


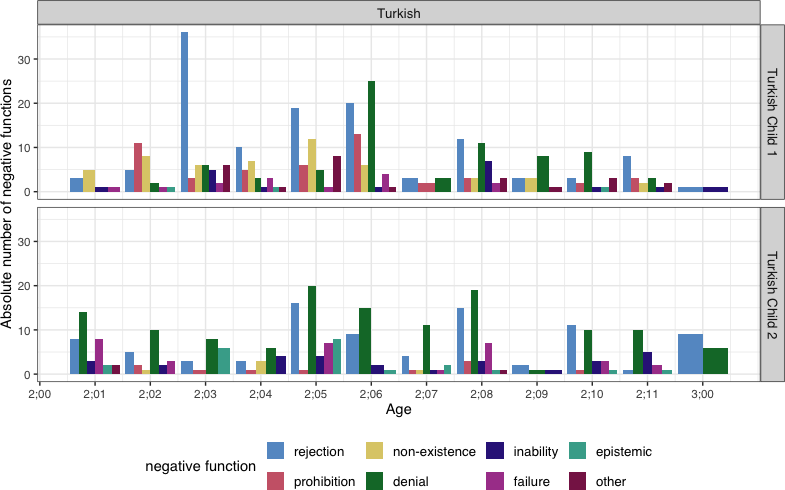


Figure 33: Turkish: The frequency of negative functions in child speech

**Reference**

Bloom, L., Hood, L., & Lightbown, P. (1974). Imitation in language development: If, when, and why. Cognitive Psychology, 6(3), 380–420.

Butters, M. (2021). Negation in four languages of Indonesia [Unpublished doctoral dissertation]. University of Colorado at Boulder.

de Swart, H. (2010). Negation in a cross-linguistic perspective. In H. Swart (Ed.), Expression and interpretation of negation (pp. 1–53). Springer.

Dryer, M. S. (2013). Negative morphemes. In M. S. Dryer & M. Haspelmath (Eds.), The world atlas of language structures online. Max Planck Institute for Evolutionary Anthropology. <https://wals.info/chapter/112>

Kridalaksana, H. (1998). Introduction to word formation and word classes in Indonesian (No. 1). Fakultas Sastra Universitas Indonesia.

Kroeger, P. (2014). External negation in Malay/Indonesian. Language, 90(1), 137–184.

Malete, E. N. (2018). Negation of subject with nominal modifiers in Sesotho. Stellenbosch Papers in Linguistics Plus, 54, 45–49.

Mermelshtine, R. (2017). Parent-child learning interactions: A review of the literature on scaffolding. British Journal of Educational Psychology, 87(2), 241–254.

Paudyal, N. P. (2015). Aspects of Chintang syntax [Unpublished doctoral dissertation]. University of Zurich.

Prieto, P., & Espinal, M. T. (2020). Negation, prosody, and gesture. In V. D. T. Espinal (Ed.), The Oxford handbook of negation (pp. 677–693). Oxford University Press.

Schikowski, R. (2013). Object-conditioned differential marking in Chintang and Nepali [Unpublished doctoral dissertation]. University of Zurich.
